# Polypharmacological Approaches for CNS Diseases: Focus on Endocannabinoid Degradation Inhibition

**DOI:** 10.3390/cells11030471

**Published:** 2022-01-29

**Authors:** Alessandro Papa, Silvia Pasquini, Chiara Contri, Sandra Gemma, Giuseppe Campiani, Stefania Butini, Katia Varani, Fabrizio Vincenzi

**Affiliations:** 1 Department of Biotechnology, Chemistry and Pharmacy, DoE Department of Excellence 2018-2022, University of Siena, Via Aldo Moro 2, 53100 Siena, Italy; alessandro.papa@student.unisi.it (A.P.); gemma@unisi.it (S.G.); campiani@unisi.it (G.C.); 2 Department of Translational Medicine, University of Ferrara, Via Fossato di Mortara 17-19, 44121 Ferrara, Italy; psqslv@unife.it (S.P.); chiara.contri@unife.it (C.C.); vrk@unife.it (K.V.); fabrizio.vincenzi@unife.it (F.V.)

**Keywords:** fatty acid amide hydrolase, monoacylglycerol lipase, endocannabinoid system, polypharmacology

## Abstract

Polypharmacology breaks up the classical paradigm of “one-drug, one target, one disease” electing multitarget compounds as potential therapeutic tools suitable for the treatment of complex diseases, such as metabolic syndrome, psychiatric or degenerative central nervous system (CNS) disorders, and cancer. These diseases often require a combination therapy which may result in positive but also negative synergistic effects. The endocannabinoid system (ECS) is emerging as a particularly attractive therapeutic target in CNS disorders and neurodegenerative diseases including Parkinson’s disease (PD), Alzheimer’s disease (AD), Huntington’s disease (HD), multiple sclerosis (MS), amyotrophic lateral sclerosis (ALS), stroke, traumatic brain injury (TBI), pain, and epilepsy. ECS is an organized neuromodulatory network, composed by endogenous cannabinoids, cannabinoid receptors type 1 and type 2 (CB_1_ and CB_2_), and the main catabolic enzymes involved in the endocannabinoid inactivation such as fatty acid amide hydrolase (FAAH) and monoacylglycerol lipase (MAGL). The multiple connections of the ECS with other signaling pathways in the CNS allows the consideration of the ECS as an optimal source of inspiration in the development of innovative polypharmacological compounds. In this review, we focused our attention on the reported polypharmacological examples in which FAAH and MAGL inhibitors are involved.

## 1. Introduction

The endocannabinoid system (ECS) is a widespread neuromodulatory network that plays an important role in the regulation of many cognitive and physiological processes by modulating neuronal activity [1]. The ECS appears to be of relevance as a therapeutic target in central nervous system (CNS) disorders, particularly in neurodegenerative diseases such as Parkinson’s disease (PD), Alzheimer’s disease (AD), Huntington’s disease (HD), multiple sclerosis (MS), amyotrophic lateral sclerosis (ALS), strokes, traumatic brain injury (TBI), pain, and epilepsy. The endocannabinoid signaling seems to be altered and hypofunctional in many neurological diseases; thus, it could represent a critical component in the control of neuroinflammation and the pathogenesis of neurodegenerative disorders [2]. These are complex diseases and are often due to the deregulation of multiple pathways, so treatment with single drugs is not effective enough. An innovative and more effective approach is polypharmacology using single molecules that can act on several targets at the same time. In this context, the ECS can be a great resource for the development of new agents because of its many connections with other neurotransmitter systems. The modulation of ECS, increasing the tone of endocannabinoids, seems to be a promising strategy for the treatment of various CNS diseases [3]. In particular, the inhibition of the endocannabinoid degrading enzymes, such as fatty acid amide hydrolase (FAAH) and monoacylglycerol lipase (MAGL), may exert therapeutic effects without inducing the adverse side effects associated with direct cannabinoid receptors stimulation [4]. Hampering endocannabinoid degradation is an attractive strategy to obtain the indirect cannabinoid (CB) receptor activation. Considering these assumptions, the present review aims to offer a glimpse of the current polypharmacological approaches involving FAAH and MAGL inhibitors, their dualism, and their polypharmacology involving other enzymes or systems with a focus on CNS disorders and especially neurodegenerative ones.

### 1.1. Polypharmacology

Polypharmacology is defined as the design or use of pharmaceutical agents acting on multiple targets. The pharmaceutical agents can be combinations of multiple drugs binding to different targets, an approach called drug combinations, or single drugs binding to multiple targets, defined as multitarget ligands [5]. Modern drug discovery has been strongly focused on the development of drugs intended to act against a specific target with high potency and selectivity. This paradigm is based on a direct cause−effect relationship between the activity of a gene product and a particular phenotype. Consequently, a pharmacological agent able to specifically modulate the activity of a deregulated protein should be able to revert a pathological phenotype [6,7,8]. Nowadays, the lack of success of highly potent and target-specific drugs in clinical development, and the limited therapeutic efficacy of single-target drugs are encouraging the design of multitarget compounds. Other than combination therapy accomplishment, the use of a single drug that modulates several targets might be therapeutically advantageous. Even if polypharmacology might be associated with compound promiscuity, it should be classified and clustered as a different category since the so-called promiscuous drugs are instead a class of compounds that show a wide spectrum of biological activities and adverse reactions [9].

Complex multifactorial pathologies, such as metabolic syndrome, psychiatric or degenerative central nervous system disorders, and cancer, cannot be effectively treated with a single-target modulation. Many lines of evidence prove that complex pathologies are often polygenic and characterized by the dysregulation of various physiological processes [10,11,12]. In a multifactorial pathological condition, the inhibition of one pathway is normally compensated by the higher activation of other pathways. This process may lead to a resistance phenotype which could require a higher drug dose with a consequent increased risk of side effects, due to the off-target modulation [9]. Therefore, these diseases could be treated more successfully with multiple target drugs [13]. The pharmacological treatment of complex diseases should aim to the modulation of the different biological targets implicated in the pathology, in order to restore the physiological balance and generate sufficient therapeutic efficacy [14,15]. Hence, multitarget drugs offer a variety of advantages: well-designed and optimally balanced multiple ligands may replace a series of drugs in combination therapy, with a subsequent decrease in treatment complexity, drug side effects, pharmacokinetic complexity, and drug–drug interactions, and patients’ adherence [8]. Moreover, modulation of multiple biological targets may increase therapeutic efficacy through synergies [16]. Multitarget drug discovery also offers economic advantages as the clinical development of a single multitarget drug requires fewer clinical trials than multiple specific drugs [9]. Multiple drugs can also be synthesized to recognize multiple binding sites on a single-target exhibiting allosteric and orthosteric modulation, such as G protein coupled receptors (GPCRs), which are implicated in multiple therapeutic areas and share structural and functional similarities that make selectivity a very difficult issue [17,18]. The majority of designed multitarget compounds either address target combinations within a protein family such as multiple kinases or GPCRs or modulate proteins involved in the same enzymatic pathways, such as the arachidonic acid (AA) cascade where the different enzymes accommodate structurally similar substrates. Still, examples of multiple ligands for markedly different biological targets demonstrate that also much more complex target combinations in terms of multitarget compound design are feasible [19,20]. Now it is accepted that many approved drugs elicit their therapeutic effects through complex polypharmacology. While current active compounds from medicinal chemistry sources with available high-confidence activity data are known to bind, on average, to only one/two targets, approved drugs have, on average, close to six known targets [21].

Single-target ligands may be affected by side effects and tissue toxicity, resulting in reduced efficacy, drug resistance, and a generally decreased quality of life for patients. Whilst multitarget drugs often show more efficacy against diseases in advanced stages [22]. Moreover, a molecule with dual activity may have a more predictable pharmacokinetic profile compared to drug combinations [23]. Data in the literature suggest that acute and delayed side effects may be more severe using drug combinations, especially when combining drugs that are not particularly selective [24]. Mixing many active principles, as in combination therapies, may result in positive but also negative synergistic effects, so this could decrease the number of useful combinations [25]. On the other hand, multitarget drugs show a minor odds of developing target-based resistance than single-target drugs [26]. Administering a multitarget-directed ligand guarantees the simultaneous presence of the molecule in all the tissues where the active principle is required to work such that the compound interacts with its multiple targets [11,27]. From the experimental point of view, developing multitarget-directed ligands might be easier because their regulatory requirements for demonstrating activity and safety are more straightforward than those for single agents. In fact, drugs combination approval is granted only after two or more agents have been assessed as single drugs and then in combination in standard therapies [28]. Drug combinations have to deal with different solubility issues, requiring adjustments in the formulation to ensure the appropriate blood level of each drug. In addition, the regulatory requirements are more complex when the agents are used in combination, since the safety profile of each drug needs to be demonstrated before clinical trials [29].

### 1.2. Endocannabinoid System, Endocannabinoids, and Exogenous Cannabinoids

**Figure 1:**
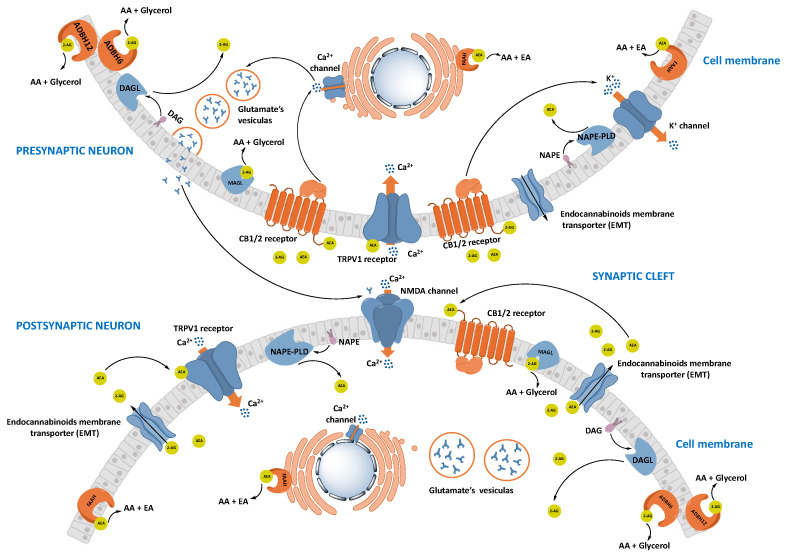
Retrograde signaling of ECS in a Glutamatergic synapse. Anandamide (AEA) and 2-arachidonoylglycerol (2-AG) are synthesized on demand in the postsynaptic neuron following an increment of Ca^2+^ concentration. AEA is synthesized starting from the membrane lipid *N*-acylphosphatidylethanolamine (NAPE) by *N*-acyl-phosphatidylethanolamine-hydrolyzing phospholipase D (NAPE-PLD) activity, while diacylglycerol lipase (DAGL) converts the diacylglycerol (DAG) in 2-AG. AEA and 2-AG move across the cell membrane through a purported endocannabinoid membrane transporter (EMT). Cannabinoid receptor type 1/2 (CB_1/2_) and transient receptor potential vanilloid 1 (TRPV1), are the main receptor targets of AEA and 2-AG on the presynaptic neuron. Activation of CB_1/2_ receptors triggers the Ca^2+^-mediated release of glutamate, in the presynaptic neuron, with subsequent activation of NMDA receptors in the postsynaptic neuron. Moreover, the transduction pathway of CB_1/2_-mediated determines the K^+^ efflux which opposes the depolarization in the presynaptic neuron. AEA is hydrolyzed by FAAH enzyme while in the 2-AG catabolism are involved MAGL, ADBH6, and ADBH12 enzymes. The outcome of this process is the inhibition of glutamatergic activity. Moreover, eCB signaling can also proceed by a non-retrograde mechanism, in which AEA and 2-AG active CB_1_ receptors or TRPV1 channel on the postsynaptic neurons.

The ECS is composed of endocannabinoids, cannabinoid receptors type 1 and type 2 (CB_1_ and CB_2_ receptors), and proteins involved in the transport, synthesis, and catabolism of the endocannabinoids (Figure 1). Most of the ECS components are multifunctional; therefore, the ECS influences and is influenced by many other signaling pathways. This is especially important to consider when evaluating the effects of ECS-targeted drugs [30].

Endocannabinoids are signaling lipids that activate CB_1_ and CB_2_ receptors [31]. The two most studied endocannabinoids are the derivatives of AA, N-arachidonoylethanolamine (AEA), and 2-arachidonoylglycerol (2-AG). Endocannabinoids are found in all tissues, organs, and body fluids studied so far. Both AEA and 2-AG are endogenous agonists of CB_1_ and CB_2_ receptors [32]. Apart from 2-AG and AEA, there are other structurally related lipids that also engage cannabinoid receptors, such as 2-arachidonoyl-glyceryl ether, O-arachidonoyl-ethanolamine, N-arachidonoyl-dopamine, and oleamide. N-oleoylethanolamine and N-palmitoylethanolamine are considered as endocannabinoids even if they lack a strong affinity for CB receptors [33,34]. Conversely, 2-AG and AEA have the potential to activate a wide range of GPCRs, nuclear receptors, and ion channels, although when considering this literature, careful examination needs to be given to the experimental design and physiological relevance of the results [35,36]. In addition, 2-AG is an important intermediate in lipid metabolism, particularly as a source of AA for prostaglandin synthesis [37]. Among the exogenous ligands in nature, there are more than 60 such as the constituents of cannabis sativa. They have closely related structures and physical properties, which make their separation difficult. In 1964, the active ingredient delta-9-tetrahydrocannabinol (Δ^9^-THC) was isolated for the first time [38]. Surprisingly, although most phytocannabinoids have now been identified and their structures are chemically related, the only major mood-altering constituent is Δ^9^-THC. Another important phytocannabinoid is cannabidiol (CBD), which over the past two decades has been found to be a potent anti-inflammatory agent, to mitigate the memory-impairing effects produced by Δ^9^-THC and to cause a variety of other effects [39].

The ECS is a pleiotropic signaling system involved in all aspects of mammalian physiology and pathology, and for this reason represents a potential target for the design and development of new therapeutic drugs. However, endocannabinoids and some of their congeners also interact with a much wider range of receptors, including members of transient receptor potential (TRP) channels, peroxisome proliferator-activated receptors (PPARs), and other GPCRs. In fact, following the discovery of endocannabinoids, lipid mediators related to endocannabinoids, which often share the same metabolic pathways, have also been identified or rediscovered [34].

#### 1.2.1. Cannabinoid Receptors

CB_1_ and CB_2_ receptors are GPCRs, which primarily couple to inhibitory G proteins [30]. Three main chemical classes of ligands activate CB_1_ and CB_2_ receptors: cannabinoids (Δ^9^-THC and to a lesser extent cannabinol) and their synthetic analogues, eicosanoids, such as AEA and 2-AG, and aminoalkylindoles [40]. Importantly, Δ^9^-THC is a low-efficacy CB_1_ receptor agonist, while for example 2-AG and most synthetic CB_1_ receptor agonists are high-efficacy agonists. [40]. CB_1_ is expressed in all brain area, in fact, it is known as being the most abundant GPCR in the mammalian brain [41]. In most brain areas, CB_1_ is expressed in the presynaptic terminals of neurons of both glutamatergic acid and gamma aminobutyric acid (GABA) which has been observed to exhibit both homodimeric and heterodimeric structures [42]. However, CB_1_ receptor can also be expressed post-synaptically and many studies have shown that it can form heterodimers in association with other GPCRs including A_2A_ adenosine receptors, dopamine D_2_ receptors, or orexin type 1 receptors [34,43]. Furthermore, CB_1_ receptor is also found in non-neuronal cells of the brain, particularly in astrocytes, where its activation promotes the release of neurotransmitters [2]. After the synthesis of the endocannabinoids in the postsynaptic sites, they travel backwards to stimulate the CB_1_ receptors on the presynaptic terminals to then be inactivated by the hydrolytic enzymes. Thus, the “on demand” production of endocannabinoids acting as retrograde signals (Figure 1), together with CB_1_ receptor-mediated activation of K^+^ and inhibition of Ca^2+^ channels, regulate the duration of synaptic activities and, subsequently, different forms of short- and long-term synaptic plasticity [44]. However, the presence of CB_1_ and TPV1 receptors ion the post-synaptic neurons suggests that eCB signaling can also proceed in a non-retrograde or autocrine manner [45]. CB_1_ receptors are also expressed in the peripheral nervous system and in almost all mammalian tissues and organs including the gastrointestinal tract, heart, liver, adipose tissue, lungs, adrenal glands, smooth and skeletal muscles, male and feminine reproductive systems, bones, and skin [34,46,47].

The function of the CB_2_ receptors is often correlated to that of the CB_1_ receptors; similarly, CB_2_ receptor subtype is a GPCR and is coupled to G_i_/G_O_ proteins. Therefore, its stimulation inhibits adenylyl cyclase (AC) activity and activates mitogen-activated protein kinase (MAPK) [34]. Unlike CB_1_ receptors, levels of CB_2_ receptors in the brain are very low and emerging studies showed that their expression is limited to specific neuronal cells and becomes abundant in activated microglia and astrocytes [17]. However, the role of CB_2_ receptors in the brain is still controversial and it remains to be conclusively established whether or not this receptor participates in affective behavior [34]. On the contrary, it is well known that CB_2_ receptors are abundantly expressed in cells belonging to the immune system such as monocytes, macrophages and B and T cells. In these cells, activation of CB_2_ receptors, among other effects, reduces the release of proinflammatory cytokines or lymphangiogenic factors [48,49]. In addition, CB_2_ receptors are also present in other peripheral organs and cell types that play a role in the immune response, including spleen, tonsils, thymus gland, mast cells, and keratinocytes as well as in the gastrointestinal system [34].

Transient potential receptor type 1 vanilloid channel (TRPV1) and G protein-coupled receptor 55 (GPR55) have been identified as other suspected cannabinoid receptors. TRPV1 belongs to a subclass of ion channels characterized by weak voltage sensitivity and non-selective permeability to monovalent and divalent cations including Mg^2+^, Ca^2+^, and Na^+^ [34]. TRPV1 activation contributes to pain transmission, neurogenic inflammation and, as suggested by more recent studies, also to synaptic plasticity, neuronal overexcitability and neurotoxicity [50,51]. TRPV1 channels are widely expressed in dorsal root ganglia and sensory nerve fibers, but also in non-neuronal cells and tissues such as keratinocytes and skeletal muscle.

GPR55 belongs to the large GPCR family and is currently considered a potential cannabinoid receptor. The endogenous ligand of this receptor is lysophosphatidylinositol, but GPR55 appears to be activated by Δ^9^-THC and some synthetic agonists of CB_1_ receptor and antagonized by the non-psychotropic phytocannabinoid cannadibiol [40,52,53]. The exact function of GPR55 is still not fully understood, but recent findings have suggested that activation of GPR55 may play an opposite role to CB_1_ receptors by increasing the release of neurotransmitters [34].

#### 1.2.2. Synthesis and Transport of Endocannabinoids

Various synthetic and degradative enzymes have been identified that dynamically regulate endogenous cannabinoid levels in normal and diseased conditions and which may be key targets for therapy. Both AEA and 2-AG are produced by the cleavage of plasma membrane phospholipids. AEA is synthesized from its precursors of AA and phosphatidylethanolamine by the sequential actions of two intracellular enzymes: N-acyltransferase (NAT) and N-acyl phosphatidylethanolamine phospholipase D (NAPE-PLD) [54]. 2-AG is formed by the hydrolysis of membrane-derived diacylglycerol by sn1-diacylglycerol lipase (DAGL) present in the membranes of neuronal dendritic spines. Expression of DAGL can also be induced in reactive astrocytes [55]. How these highly lipophilic endocannabinoids are released from the membrane into the synaptic and extrasynaptic spaces remains unclear [54]. Literature data support the existence of a putative AEA membrane transporter. However, other transporters independent from AEA uptake mechanisms have been proposed, such as caveolae-related endocytosis and facilitated diffusion driven by FAAH. Moreover, different cytoplasmic AEA-binding proteins and intracellular compartments (adiposomes) have been shown to be important for the cellular uptake of AEA [56].

#### 1.2.3. Degradation of Endocannabinoids

Inactivation of endocannabinoids occurs rapidly in vivo by cellular uptake and enzymatic hydrolysis. FAAH is primarily responsible for the degradation of AEA. Inactivation of 2-AG occurs preferentially through hydrolysis by the presynaptically localized enzyme MAGL [57,58]. To a smaller extent, 2-AG is also metabolized by FAAH, serine hydrolase/hydrolase 6 (ABDH6), serine hydrolase/hydrolase 12 (ABDH12), and cyclooxygenase (COX) 2 [59].

##### Fatty Acid Amide Hydrolase (FAAH)

FAAH is a membrane-bound protein belonging to the serine hydrolase family. This enzyme plays a significant role in the catabolism of bioactive lipids called fatty acid amides (FAA) in both the CNS and peripheral tissues [57,60]. FAAH is widely distributed throughout the body. In the rat, it was found in large quantities in the liver, followed by the small intestine, testes, uterus, kidneys, eye tissues, spleen, and possibly lungs, while skeletal muscle and heart lack this enzyme. However, any activity seen in the heart could probably be due to FAAH localized in the endothelial cells lining the blood vessels [61]. Immunohistochemical studies revealed that FAAH is widely localized in major neurons such as Purkinje cells in the cerebral cortex, pyramidal cells in the cerebral cortex and hippocampus, and mitral cells in the olfactory bulb. The enzyme is also predominantly expressed within intracellular membranes such as the outer membrane of mitochondria and the smooth endoplasmic reticulum in the neuronal somatodendritic compartment. It is found to be integrated with microsomal, mitochondrial, myelinated, and synaptosomal fractions. Thus, the expression of the FAAH enzyme varies from region to region, with the most important activities observed in the hippocampus and globus pallidus, conversely, the lowest activity was found in the brain stem. Furthermore, FAAH has cellular localization in the large neurons postsynaptic to CB_1_ receptors [57,62]. Neurochemical studies in FAAH-knockout mice showed that endogenous concentrations of AEA and other N-acylethanolamines increased 10–15 times in several brain regions including cerebellum, hippocampus, and cortex [35,38]. Fascinatingly, these elevated levels of FAA in the CNS correlated well with the CB_1_ receptor-dependent anxiolytic and analgesic effects [60,63,64]. Collectively, these findings suggest that FAAH is a key enzyme involved in FAA catabolism in vivo and demonstrated that pain pathways are impacted by a FAAH-regulated eCB tone [57]. Furthermore, the chemical inactivation of FAAH leads to an increase in neuronal transmission and/or counter controls to neuroinflammation and pain, including depression and anxiety [65,66,67]. Therefore, FAAH has been identified as a potential therapeutic target for several disorders related to the peripheral and CNS. In addition to their role in intervention in neuropathic pain and neuroinflammation, FAAH inhibitors have also been found to counterbalance the addiction and related effects of nicotine [68,69]. These activities take place without any changes in weight gain, motility, sleep, or other side effects typically seen with direct CB_1_ receptor agonists [57]. The main action of FAAH inhibitors is to increase the endogenous levels of AEA thus extending the duration of its biological effect, representing a potential therapeutic strategy for various diseases [70].

##### Monoacylglycerol Lipase (MAGL)

MAGL is a soluble membrane-associated enzyme, which belongs to the superfamily of serine hydrolase [61]. MAGL preferentially hydrolyzes monoacylglycerols to glycerol and fatty acids with no positional preference for sn-1 (3) or 2-monoacylglycerols (MAG) [49]. MAGs are always short-lived lipids, which may come from both intra- and extracellular compartments. One of the important MAGs is the endocannabinoid 2-AG, which can be degraded into AA and glycerol [61]. In most tissues, including the brain, more than 80% of the hydrolytic activity of 2-AG is prevented by the inhibition of MAGL, suggesting the dominant role of MAGL for the degradation of 2-AG [71,72]. Other studies indicated that glycerol esters of prostaglandins, the mediators of inflammation, could also be hydrolyzed by MAGL [73]. More recently, MAGL has been identified to hydrolyze the ethyl esters of fatty acids that are generated in response to alcohol consumption [74]. MAGL is highly expressed in the brain, liver, adipose tissue, intestines, and others, and this was demonstrated by both genetic and pharmacological inhibition of MAGL in mice. In the brain, MAGL is expressed in neurons, astrocytes and oligodendrocytes and, to a lesser extent, in microglia [61,62]. AA, the metabolite of 2-AG and AEA, is the main precursor for pro-inflammatory synthesis of prostaglandins. Since the physiological levels of 2-AG are much higher than those of AEA, interest has been renewed in the search for inhibitors for this enzyme [42]. Nomura et al. demonstrated that MAGL was the main AA providing enzyme for the biosynthesis of eicosanoids in some tissues [75]. Furthermore, many studies, both genetic and pharmacological, have demonstrated the important role of MAGL in the regulation of endocannabinoid and eicosanoid signaling pathways [76,77,78]. In fact, the pharmacological inactivation of MAGL reduces the hydrolytic activity of 2-AG by 80% in most tissues including the brain. Therefore, MAGL is considered to be a promising therapeutic target for the treatment of various disorders, including neurodegenerative ones, inflammation, metabolic diseases, and even cancer [61]. Cannabinoids have been used as analgesics for quite a long time and only recently has the ECS been linked to inflammation [39,79]. Inflammatory processes are always associated with multiple neurodegenerative disorders. Furthermore, pain and inflammatory processes are considered a hallmark of neurological diseases, including AD, PD, MS, and stroke [80]. CB_1_ and CB_2_ receptors agonists and COX inhibitors have been shown to have beneficial effects on various inflammatory diseases. However, the use of COX 1–2 inhibitors has been limited because they can cause gastrointestinal and cardiovascular damage [81,82]. MAGL was discovered to reduce AA and prostaglandin levels in specific tissues, suggesting its potential as a therapeutic target for inflammation. In mice treated with lipopolysaccharides (LPS), administration of a MAGL inhibitor reduced the formation of prostaglandins and pro-inflammatory cytokines [75,83]. Inhibition of MAGL produced neuroprotective effects in animal models of PD and MS [84,85,86]. 2-AG accumulation deriving from MAGL inhibition leads to the activation of cannabinoid receptors. However, these neuroprotective responses appear not to be guided through the cannabinoid receptor-dependent pathway, but by lowering pro-inflammatory eicosanoids. Attenuated neuroinflammatory responses in animal models were not reversed on cannabinoid receptor antagonists, indicating that the protective effects observed were mainly due to decreased levels of prostaglandins and cytokines in the brain. However, chronic inhibition of MAGL inducing functional desensitization of the cannabinoid system could also contribute to the neuroprotective response [61].

### 1.3. Interconnections between ECS and Neurotransmitter Systems

The ECS is closely correlated with other systems and cooperates to regulate many cognitive and physiological processes, primarily via controlling both GABAergic and glutamatergic neurons in the synaptic terminals of many brain areas involved in emotional behaviors included social and cognitive activity [31,87]. Thanks to the interconnections with other systems, the ECS is arousing new interest for many neurological and neuropsychiatric diseases [88]. An important interaction has been found between ECS and the dopaminergic system; dopamine is an important neurotransmitter in the brain which plays a major role in learning, motivation and reward, emotion, executive functions, and motor control [89,90,91]. ECS is a filter of afferent input that acts locally at midbrain and terminal regions to shape how incoming information is conveyed onto dopamine neurons and to output targets [92]. Numerous studies seem to support the idea that endocannabinoids regulate dopamine by means of other neuronal subpopulations, such as GABAergic and glutamatergic neurons [93,94,95]. The dopamine system also has a critical role in the development of various substance addiction and withdrawal. The most common substances of abuse such as cocaine, amphetamine, morphine, nicotine, and alcohol, increase extracellular dopamine concentration in the striatum; thus, the ECS modulation can be beneficial as a novel therapeutic strategy in various scenarios of substance withdrawal and abuse [96]. Interaction between ECS and the dopaminergic system is very intricate and complex and regards all the neurobehavioral aspects regulated by dopamine, including motivation and reward underlying the basic survival instinct to a higher hierarchy of needs, such as self-actualization of an individual [97]. Endocannabinoids also interact with the serotoninergic system, the behavioral effects of serotonin and endocannabinoids activity have been widely reported, including the regulation of emotional states, stress homeostasis, cognitive functions, food intake, and sleep. The distribution pattern of the serotoninergic system and the ECS in the brain display a strong overlap; several studies report a functional interplay and even a tight interdependence between endocannabinoids and serotonin signaling [98].

Moreover, the ECS has an important crosstalk with the cholinergic system. It has been reported that activation of the muscarinic M_1_ receptor tonically inhibits endocannabinoid release at glutamatergic synapses through suppression of channel-mediated Ca^2+^ currents [99]. In the striatum, acetylcholine (ACh), acting on muscarinic M_1_ receptors, constitutively upregulates the depolarization-induced release of endocannabinoids from medium spiny neurons. The released endocannabinoids cause transient suppression of inhibitory synaptic inputs to medium spiny neurons through acting retrogradely onto presynaptic CB_1_ receptors [100]. Thus, muscarinic system regulation of striatal output and endocannabinoids release may contribute to motor control.

Numerous studies have shown that GPCRs, including cannabinoid receptors, can exist and function as higher-order dimers or complexes [101,102]. This oligomerization can affect receptor signaling, receptor trafficking, and ligand binding. The physiological relevance of this dimerization has not yet been fully established for cannabinoid receptors; however, the presence of homo- and heterodimers cannabinoids in specific tissues has been intensively reported in recent years [36]. For the CB_1_ receptor, the existence of heteromers under certain physiological conditions has been suggested with the serotonin, angiotensin, opioid, GPR55, somatostatin, orexin receptors dopamine, and adenosine receptors among others [103,104,105,106,107,108,109,110,111,112,113,114,115]. Although the CB_2_ receptor has been studied less, recent research has revealed that it can form heterodimers with CB_1_ receptor, with GPR55 receptor, with the 5-HT_1A_ serotonin receptor, or with the chemokine receptor CXCR4 [116,117,118,119,120]. The expression of these heterodimers has been associated with several pathologies. For example, CB_2_-CXCR4 and CB_2_-GPR55 dimers have been associated with cancer progression, while CB_1_-A_2A_ and CB_1_-D_2_ heteromers have been suggested to have physiological implications in neurodegenerative disorders such as AD, PD, epilepsy, autism, but also in neuropsychiatric pathologies such as anxiety, depression, and psychotic disorders [36,88].

#### ECS Relevance to CNS Diseases

The ECS is deeply involved in the development of the CNS and the regulation of neuronal cell fate through the dynamic expression of endocannabinoids during neurogenic processes [121]. In prenatal development, the levels of AEA and 2-AG vary considerably, while FAAH is expressed in glial cells during late pregnancy and in the postnatal period. In vivo, astrogliogenesis is driven by the distribution patterns of FAAH and endocannabinoids, suggesting a predominant role of the ECS in neuronal progenitor cell differentiation [122]. For example, AEA in cooperation with brain-derived neurotrophic factor (BDNF), a principal pro-differentiating neurotrophin, induces the migration of GABA-containing interneurons in the embryonic cortex [123]. During the development of the CNS, there is an extremely fine-tuned regulation of the balance between proliferation and programmed death of progenitor cells. This ensures that the right number of neuronal cells are generated. Various studies have shown that the ECS is involved in the regulation of engagement, survival, and synaptic connectivity in the developing brain, probably through the involvement of ERK 1/2 and the inhibition of Ras-associated protein-1 (Rap1) and serine/threonine protein kinases B-raf [124,125].

Considering the multiple roles of ECS in the regulation of dopaminergic system development, GABA interneurons differentiation, and neuronal development and plasticity, this system represents a promising target in the treatment of CNS diseases [126]. In fact, it has been demonstrated that ECS is impaired in a number of neuropsychiatric pathologies, particularly in disorders such as anxiety and depression. In these conditions a possible strategy could be to inhibit endocannabinoid degradation by synthetic molecules, thus restoring the altered turnover of endocannabinoids and indirectly activating CB receptors only in the tissues where the alteration occurs, avoiding the side effects of direct receptor activation [127]. Several studies have demonstrated that the endocannabinoid content in the tissues and serum of patients with depression shows marked variation compared to those of healthy individuals. CB_1_ receptor expression and 2-AG levels have been found to be significantly reduced in the hippocampus as a result of chronic unpredictable stress, which is thought to mimic the behavioral and endocrine changes that promote the development of human clinical depression [128,129,130]. An interesting study on the changes in the ECS revealed that the serum level of 2-AG is significantly decreased in patients with major depression, while in patients with mild depression, the serum content of AEA and 2-AG showed a tendency to increase [131]. This implies that the ECS could be interpreted as a regulator in depressive disorders. There is a strong negative correlation between serum AEA level and anxiety symptoms in patients with major depressive disorder [3]. This evidence supports interaction between ECS and depression/anxiety. Indeed, clinical studies reported that genetic polymorphisms that decrease FAAH activity and increase baseline AEA levels are associated with reduced anxiety. Similar outcomes were reported in people treated with FAAH inhibitors, which also showed facilitated consolidation of emotional memories, and protection against negative emotional consequences following exposure to stress [132,133].

In the hippocampus of AD patients 2-AG levels were increased, this was also confirmed in rodent models [134]. Endocannabinoids increase may offer neuroprotection acting on CB_1_, receptors and inhibiting the inflammatory microglial response through activation of CB_2_ receptors. In fact, CB_2_ agonists can reduce TNF-a and nitric oxide production by microglia and macrophages, and stimulate their phagocytosis of amyloid β [135,136,137]. Moreover, CB_2_ receptors and FAAH were selectively overexpressed in neuritic plaque-associated glia in the AD brain, especially in reactive astrocytes and activated microglial cells [3,138]. A CB_2_ receptor agonist demonstrated the ability to stimulate the natural removal of amyloid β in frozen human tissue sections and inhibit the synthesis of pathogenic peptides [136]. CB_2_ receptor and FAAH expression patterns are closely related to amyloid β deposition, suggesting that they may play a regulatory role in microglial cell function in AD-associated pathological changes. The expression of CB_1_ receptor in different brain regions of AD patients remains controversial; however, research has indicated that CB_1_ receptor activity is contingent on the clinical period of AD, with increased CB_1_ receptor activity found in the early stage of AD and reduced levels found in later stages [139]. Studies in animal models of amyloid β neurotoxicity report an increase in 2-AG in the hippocampus thanks to its augmented synthesis [134]. These findings are in agreement with a previous report on elevated DAGL enzymatic activity in the hippocampus of patients with AD, suggesting a tissue-selective enhancement of 2-AG levels [140].

ECS is also involved in PD, actually, CB_1_ receptors are highly distributed in the basal ganglia and exert complex regulatory effects on some important neurotransmitters, playing a role in anti-excitatory neural toxicity and neuroprotection [141]. Studies have reported that the MAGL inhibition produces neuroprotective effects in animal models of PD leading to the accumulation of 2-AG and the activation of cannabinoid receptors [84,85]. However, these neuroprotective responses appear not to be dependent on the cannabinoid receptor pathway, but on the decrease in pro-inflammatory eicosanoids. Attenuated neuroinflammatory responses in PD animal models were not reversed by cannabinoid receptor antagonists, indicating that the protective effects observed were mainly due to decreased levels of prostaglandins and cytokines in the brain. However, chronic inhibition of MAGL, which induces functional desensitization of the cannabinoid system, could also contribute to the neuroprotective response [58].

Although neurodegenerative diseases have different pathogenesis and progression, there are some common features such as hypofunctionality or deregulation of the ECS. Changes in endocannabinoid levels and CB receptors are induced by the neuroinflammation that is common to these diseases [54]. Activation of CB_2_ receptors has the task of limiting the activation of microglial cells, but unfortunately this response is not sufficient to counteract the inflammatory state and prevent neuronal damage. In order to increase the innate anti-inflammatory response of the brain the treatment with inhibitors of endocannabinoids catabolic enzymes may be very useful.

Recent studies also reported that repeatedly eliciting seizures in the amygdala caused a long-term increase in anxiety and impaired fear memory retention, which was associated with a GABA/glutamate presynaptic activity imbalance and to an alteration of synaptic plasticity in male rats [142]. This was associated with a reduced AEA signaling in the amygdala, leading to a loss of tonic inhibition on the glutamatergic synapses and a loss of phase control on the GABAergic synapses, thus causing emotional alterations. Furthermore, FAAH inhibition normalized both behavioral and electrophysiological dysfunction in rats that had repeated seizures [142,143,144]. These data suggest that FAAH inhibitors may have a potential two-pronged therapeutic approach in seizure-related disorders, being able to reduce seizure severity themselves as well as relieving the development of comorbid emotional changes. This dual treatment approach offers a whole new avenue in the treatment of seizure-related disorders [142,145]. Another study has shown how indirect activation of the CB_1_ receptor, using MAGL inhibitors, has brought benefits for epileptic seizures. During the convulsive activity, the increased levels of 2-AG probably mainly activated CB_1_ receptors on the main neurons, suppressing glutamate release. This could represent the key mechanism for controlling neuronal excitability during seizure activity [146].

Many lines of evidence suggest that abnormal endocannabinoid signaling may be involved even in autism spectrum disorder (ADS). First, endocannabinoids play an important role in neurological development, which is also influenced by exogenous cannabinoids. Importantly, these lines of research have not addressed whether deficient endocannabinoid signaling contributes to the main component of ASD: social harm [147]. Limited literature suggested a role for endocannabinoid signaling in normal social behaviors. Genetic removal of CB_1_ receptors alters social interactions in mice in a context-dependent manner, which may be related to social anxiety and/or cognition [148,149]. In contrast, genetic removal of FAAH in rats increases social interactions, and inhibition of FAAH promotes social play in rats [150,151]. Thus, the bidirectional modulation of social behavior is likely to depend on the dose and identity of the circuits involved. A signaling mechanism in male mice has recently been identified by which oxytocin drives anandamide-mediated endocannabinoid signaling to control social reward [152]. A further study found that social impairment is corrected in two distinct mouse models by increasing anandamide activity through FAAH inhibition. This provided new insights into the role of endocannabinoid signaling in social behavior and validated FAAH as a new therapeutic target for the social harm of ADS [145,147].

Furthermore, individuals who experience life-threatening psychological trauma are at risk of developing a series of chronic neuropsychiatric pathologies that include generalized anxiety, depression, and drug addiction. The ECS has been implicated in the modulation of these responses by regulating the activity of the amygdala and the hypothalamic–pituitary–adrenal axis. It has been demonstrated that MAGL inhibition suppresses anxiety-like behavior triggered by the exposure to a life-threatening stimulus, an effect prevented by CB_1_ receptor blockade. Thus, the pharmacological strategies aimed at enhancing 2-AG signaling at CB_1_ receptors may offer a novel therapeutic approach to the treatment of pathological sequelae of psychological trauma, such as post-traumatic stress disorder and substance abuse [153].

Considering all the data in the literature, it can be stated that the ECS has a pivotal function in neuronal development along with a widespread role in neurodegeneration. Thus, from a clinical point of view, it represents a target for the development of ECS modulating compounds useful in the treatment of neuropsychiatric and neurodegenerative disorders.

## 2. Multitarget Approaches Involving FAAH and MAGL Inhibitors

### 2.1. Dual FAAH-MAGL Inhibitors

In the last few years, academic and industrial efforts have been strongly focused on the development of selective FAAH or MAGL inhibitors with potential therapeutic application in several diseases such as MS, epilepsy, neuropathic pain, and chronic pain disorders [86,144,154,155,156]. However, the simultaneous inhibition of the two main ECS catabolic enzymes also appears as a promising therapeutic strategy. Increased levels of 2-AG and AEA deriving from dual FAAH and MAGL blockage were effective in reducing inflammatory pain without inducing cannabinoid-like effects [157]. The contribution of ECS in pain modulation seems to be effective using dual FAAH/MAGL inhibitors which determined antinociceptive effects in a visceral pain model [158]. This multitarget approach also produced relevant beneficial effects in the reduction of withdrawal signs in morphine-dependent mice [159]. Despite the above considerations and the potential benefits, the polypharmacological approach concerning dual FAAH/MAGL inhibitors remains not particularly explored. Two relevant examples of hybrid FAAH/MAGL inhibitors, JZL195 and SA-57 (see Figure S1) were reported in literature and were in deep studies in several model of different diseases. Long et al. with the developed of JZL195 reported the first example of dual FAAH/MAGL inhibitor [76]. This compound showed IC_50_ values of 13 nM and 19 nM against FAAH and MAGL, respectively, acting as a covalent inhibitor. It was designed starting from the *N*-carbonyl piperidine moiety present in both MAGL selective inhibitor JZL184 (IC_50_ *r*FAAH = 4 M, IC_50_ *r*MAGL = 4 µM, Figure S1) and FAAH selective inhibitors PF-622 (IC_50_ *r*FAAH = 33 nM, Figure S1) and PF-3845 (IC_50_ *r*FAAH = 16 nM, Figure S1) (3–20 mg/kg i.p., 4 h) showed dose-dependent FAAH and MAGL inactivation, resulting in high levels of AEA e 2-AG in vivo. These data were comparable with those observed using selective FAAH and MAGL inhibitors, respectively, PF-3845 and JZL184 [160]. Moreover, pharmacology studies in vivo were performed to investigate the role of AEA e 2-AG in specific processes. Hypomotility and hyperreflexia were observed after treatment with JZL195 and JZL184 but were not detected when PF-3845 was administrated, indicating that the AEA-FAAH pathway is not involved in these behaviors [160]. In this context, other studies were performed, comparing the effects of JZL195 and JZL184 (5, 10 and 30 mg/kg, respectively) on endocannabinoid transmission and locomotor activity in male Wistar rats [161]. JZL195 reduced motor activity in a dose-dependent manner at all doses tested while two gave the same effects only at 30 mg/kg. One hour after compounds injection AEA and 2-AG levels were measured in nucleus accumbens, caudate-putamen, hippocampus, and prefrontal cortex. JZL195 significantly increased both AEA and 2-AG levels in all brain regions and JZL184 elevated 2-AG levels only. Although pretreatment with 1 mg/kg of a CB_1_ antagonist SR141716 (Figure S1), completely reversed the effects of JZL195, while SR141716A did not show antagonizing effects in the motor disorders mediated by JZL184. These results indicated that the hypo locomotive effects of derivate JZL184 are not correlated with 2-AG increased levels but appear mediated by a CB_1_-independent mechanism [161]. On the other hand, antinociceptive effects were more evident in mice treated with the dual inhibitor JZL195, highlighting that both 2-AG and AEA can regulate pain sensation [160]. In this context, Greco et al. also evaluated the antinociceptive activity of JZL195 in male Sprague-Dawley rats, a specific model for migraine [162]. Compound **1** (3 mg/kg, i.p.) was administrated two hours after nitroglycerin (NTG, 10 mg/kg, i.p.) pretreatment, needed to induce migraine and hypomotility. Two more hours after, rats were subjected to open field test and orofacial formalin test to evaluate behavior and motility improvement and JZL195 antinociceptive effects, respectively. In the open field test, coadministration of NTG and JZL195 did not show relevant changes in locomotor activity and in explorative behavior in rats. In the orofacial formalin test, animals were treated with formalin injection (1.5%, 50 µL) and the nocifensive response was recorded for 45 minutes. JZL195 was found to reduce formalin pain in NTG + JZL195 group. To investigate the involvement of CB_1_ and CB_2_ during the orofacial formalin test, it was also conducted in the presence of JZL195 together with CB_1_ antagonist AM251 1 mg/kg, i.p., Figure S1) or CB_2_ antagonist AM630 (1 mg/kg, i.p., Figure S1) administrated two hours after NTG injection. AM251 was able to reverse the analgesic effects CB_1_ mediated, while no significant differences were found during the test in the presence of compound AM251. These data showed how derivate JZL195 determines antinociceptive effects through CB_1_ receptors [162]. The activity of JZL195 was also measured in murine inflammatory pain models by Anderson et al. In this work, C57BL/6 mice were treated with compound **1** (30 mg/kg), a selective FAAH inhibitor URB597 (IC_50_ *r*FAAH = 33 nM) (reported in Figure S1) (10 mg/kg), a selective MAGL inhibitor JZL184 (15 mg/kg), and with a non-selective CB_1/2_ agonist WIN55212 (Figure S1, 3 mg/kg). Dual inhibitor JZL195 reduced inflammation and induced allodynia at lower doses if compared with the dose that produced cannabinoid side effects. The maximal reduction allodynia produced by the dual inhibitor was even greater than the pharmacologic effect exercised by mono-target inhibitors JZL184 and URB597. Even though JZL184 and WIN55212 showed comparable therapeutic effects, the pan CB_1/2_ agonist produced side effects at the same doses at which allodynia was also detected [157]. Analogues results were obtained by Barnes et al. in a study conducted to define the right therapeutic ratio for compound JZL184 in a murine model for neuropathic pain. These convergent results proved that derivative JZL184 owns a preferable therapeutic window compared to WIN55212, appointing the dual FAAH/MAGL inhibitors as efficacious tools for the treatment of pain [163].

In the catalepsy tests, monotherapy with WIN55212 or PF-3845 did not show relevant effects while JZL195 and the coadministration of JZL184 and PF-3845 led to cataleptic conditions which were less serious if compared to those mediated by CB_1_ agonists [160]. Long et al., also examined how the activity of 2-AG and AEA, could influence drug abuse. Mice were treated with the CB_1_ agonist THC (5.6 mg/kg) and then with selective or dual FAAH–MAGL inhibitors. The “marijuana-like” effects were detected following dual FAAH/MAGL blockage, but not afterwards selective FAAH or MAGL inhibition [160]. Though, dual inhibition of ECS catabolic enzymes showed anti-withdrawal effects in morphine-dependent mice [159]. In this study, Ramesh et al. tested the therapeutic combination of low doses of compound 2 co-administered with high doses of FAAH inhibitor PF-3845. They compared these results with the use of *O*-hydroxyacetamide SA-57 (Figure S1) a dual FAAH/MAGL inhibitor (IC_50_ *m*FAAH = 1.0 nM, IC_50_ *m*MAGL = 410 nM) developed by Sanofi-Aventis, which represented a prototype of innovative compounds as pharmacological tools for CNS disorders [159,164]. The combination of partial MAGL and complete FAAH inhibition, induced by SA-57 (1, 2.5, 5 mg/kg), blocks a wide spectrum of morphine withdrawal signs. These therapeutic benefits resulted to be discernible from THC-like side effects, evident only at high doses of SA-57 (12.5 mg/kg i.e), that characterized the dual FAAH/MAGL inhibition [159,160]. Since several clinical liabilities concern opioid prescriptions for pain treatment, Wilkerson et al.demonstrated the SA-57 intrinsic antinociceptive effects and its ability to augment morphine-induced antinociception and to reduce heroin seeking behavior in male C57BL/6J mice [165]. At the doses of 1.79 mg/kg and 1.12 mg/kg for compound **10** and morphine, respectively, a complete reversion of chronic constriction injury-induced allodynia was detected, without displaying any cannabimimetic effects. Moreover SA-57 (1, 2.5, 5 mg/kg) decreased heroin self-administration at all doses tested [165]. Overall, these results indicate that dual FAAH and MAGL inhibition represent a valid therapeutic strategy to decrease opioid doses in clinical pain control and for the treatment of opioid dependence [159,165]. Based et al. compared the anxiolytic profile of JZL184 (5, 8, and 10 mg/kg i.p.), the selective FAAH inhibitor PF-3845, (0.1, 1, and 10 mg/kg i.p.) and JZL195 (5, 10, and 40 mg/kg i.p.) in C57BL/6J mice. Animals were subjected to restrain tests, foot shock stress test, light-dark box test, novelty induced hypophagia test, elevated zero maze test, open field test, Morris water maze test, and Barnes-maze test after pharmacological treatment. In these conditions, JZL184 did not show relevant anxiolytic effects while JZL184 was more efficacious than both PF-3845 and the dual inhibitor JZL195 [166]. A key role of brain-derived neurotrophic factor (BDNF) was recently demonstrated in the pathogenesis of major depressive disorders [167]. On these bases, Dong et al. examined the role of ECS in depressive behavior, measuring AEA, 2-AG, and BDNF levels after the administration of JZL195 in WKY female rats (a rat model of depression condition) to evaluate its antidepressant activity [168]. JZL195 treatment (3 mg/kg, i.p. for 7 days) of WKY rats enhanced BDNF, 2-AG, and AEA levels in ventral striatal tissue 24 h post-injection when compared with vehicle-treated rats. The beneficial effects of these treatments were observed in a forced swim test, in which WKY rats showed a greater immobility that was in part reversed 48 h post-injection of JZL195. In the same work, simultaneous FAAH/MAGL inhibition increased sucrose intake in WKY rats indicating an increase in reward sensibility endocannabinoid-mediated [168]. The effects of compound JZL195 on mice memory model was studied by Wise et al. This dual FAAH/MAGL inhibitor was tested at 20 mg/kg in FAAH^−/−^ and FAAH^+/+^ mice, involved in Morris water maze test. Mice spent more time in the target zone when treated with the vehicle compared to the THC (10 mg/kg) or JZL195 treated group. These data demonstrated that JZL195 induced alteration in short-term memory, producing THC-like effects in FAAH^−/−^ and FAAH^+/+^ mice [169]. Since the spinal cords are involved in the modulation of itch, Yesilyurt et al. investigated the role of FAAH, MAGL, and dual FAAH/MAGL inhibitors as antipruritic agents. They examined and compared the dose-related antipruritic effects of systemic or intrathecal (i.t.) administration of PF-3845 (5, 10 and 20 mg/kg, i.p.; and 10 μg, i.t.), JZL184 (4, 20, and 40 mg/kg, i.p.; 1, 5, and 10 μg, i.t.), and JZL195 (2, 5, and 20 mg/kg, i.p.; 1, 5, and 10 μg, i.t.) on serotonin (5-HT)-induced scratching model, using Balb-C mice. Results suggested that systemic and i.t. administration of all compounds produce dose-dependent antipruritic effects, designating FAAH, MAGL, and dual FAAH/MAGL inhibitors as promising therapeutic agents for the treatment of pruritic diseases [170].

A suitable drug candidate should possess an appropriate balance between potency and drug-like properties [171]. Apart for derivatives JZL195 and SA-57, whose activity was intensely studied from a pharmacological standpoint; to date, we could not retrieve other relevant pharmacological studies on dual FAAH/MAGL inhibitors, probably due to the difficulties in achieving the right balance among potency and therapeutic window for this class of compounds. However, in the recent literature some examples of dual FAAH/MAGL inhibitors can be found. In 2012, Cinsneros et al. developed a new series of reversible dual FAAH/MAGL inhibitors [172]. Docking and SAR studies were performed to rationally design and synthesize biphenyl and 4- phenylbenzyl ester derivatives, represented as general structures A and B (Table 1), considering different parameters such as length of the spacer and modification in the biphenyl moiety. Final compounds were tested against *hr*MAGL in vitro, and their ability to block 2-oleoylyglycerol (2-OG, an alternate MAGL substrate) and AEA hydrolysis in brain homogenates and in membrane fractions, respectively, was also evaluated. Biphenyl oxirane derivate (±) **1** (Table 1) (*hr*MAGL IC_50_ = 4.1 µM, rat brain FAAH IC_50_ = 5.1 µM) and 4-benzylphenyl derivate (±) **2** (Table X) (*hr*MAGL IC_50_ = 16 µM, rat brain FAAH IC_50_ = 0.28 µM) both tested as racemic mixture, showed a good inhibition profile against FAAH and MAGL enzymes [172]. Resolution of the enantiomeric mixture of **1** gives the corresponding enantiomers (*R*)-**1a** (Table 1) and (*S*)-**1b** (Table 1), which showed an inhibitory potency very close to the racemic **1** against 2-OG, while both enantiomers were less active against *hr*MAGL. Compound **1a** maintained a good efficacy against FAAH (IC_50_ = 3.9 µM) whilst compound **1b** was less potent. Enantiomers of compound **2** showed remarkable differences compared to the racemic mixture inhibition. (*R*)-enantiomer **2a** (Table 1) was more active than (±) **2** against *hr*MAGL, while (*S*)-enantiomer **2b** (Table 1) resulted inactive in the same test. Although, the inhibition on the AEA hydrolysis was not influenced by stereochemistry of the compounds [172]. Moreover, for compounds (±)-**1** and **2b** the inhibitory mechanism was studied, by a dilution experiment, confirming that they behave as reversible dual FAAH/MAGL inhibitors [172].

In 2014, Korhonen et al. developed a large set of compounds, in which different leaving groups were combined with two different scaffolds obtaining FAAH, MAGL, or dual FAAH/MAGL inhibitors [173]. Replacing the para-nitrophenol moiety of phenoxyphenyl piperazine derivative JZL195 with various leaving groups, a first set of derivatives was obtained. Benzotriazole and triazolopyridine derivatives (**3a** and **3b,** Table 2) showed a dual FAAH/MAGL inhibition profile, while the *O*-hexafluoroisopropanolyl carbamate **3c** (Table 2) was proved as a selective MAGL inhibitor; these data were in agreement with what was reported by Chang et al. [174]. The 4-phenoxy substituted imidazole urea **3d** (Table 2) was 4-fold more potent on FAAH (IC_50_ = 3.4 nM) maintaining a modest MAGL inhibition (IC_50_ = 660 nM). Except for the 1,2,4-triazole derivative **3e** (Table 2), other leaving groups tested on methylene-3,4-dioxyphenyl piperidine scaffold, did not show relevant results. This study outlined triazole heterocycles as the best performing moiety to engage FAAH and MAGL enzymes since their conjugate acids pKa values guarantee effective interaction with the catalytic serine of both targets. In fact, for selectively engaging the MAGL enzyme, the pKa of the leaving group should be between 8 and 10. These data are in line with the IC_50_ values of **3c** and **3e** where the pKa values of the leaving groups pf the hexafluoroisopropanol (HFIP) and triazole are 9.3 and 10.0, respectively. However, diverse leaving groups are tolerated by the FAAH enzyme, as highlighted by good IC_50_ values of benzotriazole, triazolopyridine, and imidazole ureas (**3a**, **3b** and **3d**) [173].

Through structure–activity relationship (SAR) and molecular docking studies, Brindisi et al.identified the structural requirements for a dual FAAH/MAGL inhibition, developing a small library of compounds (**4a**–**d** represented in Table 3) that showed a balanced inhibition profile against both enzymes [175]. The appropriate combination between pyrrloquinoxaline-based scaffold and the piperazine carboxamide/carbamate moiety was explored to identify compounds characterized by suitable size and geometrical shape for fitting FAAH and MAGL binding pocket. Developed compounds showed IC_50_ values in the nanomolar range against both enzymes and these biological data were supported by detailed docking studies performed on the lead compound **4a**. The latter occupied the binding pocket of the FAAH enzyme, and it was involved in key π–π interactions where the triazole and the tricyclic portion engaged F192 and F432, respectively. In the MAGL active site, the carbonyl group of **4a** established an H-bond with the backbone of A51 in the oxyanion hole, while the triazole moiety forms a double π–π staking with H269 and H121. The pyrroloquioxaline system gives a series of hydrophobic contacts. Moreover, carbonyl group of **4a** establish an H-bond with catalytic serine S122 and S241 for MAGL and FAAH, respectively, and it was found at the optimal distance (<2 A°) to generate a tetrahedral intermediate in both targets. In the developed compounds combination of the pyrroloquioxaline scaffold and 1,2,4 triazole leaving group guarantees an excellent dual FAAH/MAGL inhibition [175].

### 2.2. FAAH Polypharmacology

As previously mentioned, polypharmacology provides a smart contribution in exerting super-additive effects at the biological level. In line with this consideration, some new molecular entities have been reported as dual FAAH/MAGL inhibitors, opening the way for a new storytelling in medicinal chemistry. However, the multiple ECS neuro-connections provide the attractive opportunity to modulate the activity of FAAH or MAGL enzymes simultaneously to other relevant neuro-transmitting or enzymatic systems by using a polypharmacological approach. Considering the remarkable role of AEA in neuromodulation, FAAH enzyme was also selected in the last year as a promising target in other polypharmacological applications. The involvement of CB_1_ receptors and COXs enzymes in the pain modulation led to the development of dual FAAH/COXs inhibitors as possible therapeutic options for pain treatment [176,177]. Activation of CB_2_ receptors by epoxidized fatty acids (EpFAs) together with the AEA effects on CBs receptors laid the rational basis for the development of multitarget FAAH/Soluble epoxide hydrolase (sEH) inhibitors, as antinociceptive agents [178]. The neuroprotective properties of FAAH inhibitors and the therapeutic efficacy of anti-cholinesterase agents were combined obtaining hybrid FAAH/COXs inhibitors, potentially useful for the treatment of AD [179]. Simultaneously targeting the ECS and the dopaminergic system could represent an innovative strategy to fight drug abuse and the abstinence response [180]. Moreover, for the treatment of glaucoma the use of dual FAAH and melatonin receptors antagonists resulted in a new viable strategy [181].

All these therapeutic options are described below from a medicinal chemistry point of view, analyzing the keys pharmacophoric elements needed to simultaneously engage the selected targets and the therapeutic possibilities offered by these polypharmacological approaches.

#### 2.2.1. FAAH/COX Dualism

Nonsteroidal anti-inflammatory drugs (NSAIDs), which exert their action by inhibiting COX-1 or -2, are widely used treatments for acute and chronic pain [182]. Both CB_1_ receptors and COXs are involved in the pain perception, monitoring endogenous levels of arachidonoyl-based mediators [183]. In this context, clinical studies demonstrated that NSAIDs-mediated analgesic effects can be enhanced co-administrating FAAH inhibitors, indicating that the dual FAAH/COX inhibition is a valuable option in pain treatment [176,177]. This polypharmacological approach combines synergic interactions between FAAH/COX blockage, reduces COX-mediated side effects, and prevents clinical risks derived from drug–drug interaction [184,185]. In 2012, Bertolacci et al. resolved the crystal structure of FAAH in complex with the COX-1-2 inhibitor 2-(6-chloro-9H-carbazol-2-yl)propanoic acid carprofen (COX-1 IC_50_ = 22.3 ± 6.6 µM and COX-2 IC_50_ = 3.9 ± 1.0 µM, Table 4) at 2.5 Å resolution [186]. The propionic acid moiety of caprofen, which is ionized at the pH used to crystalize the complex (pH 7.4), forms an H-bond with the Trp531. On the other hand, the carbazole ring occupies the membrane access channel (MAC) of FAAH interacting whit hydrophobic amino acids. This binding mode was confirmed by the evaluation of carprofen activity against recombinant wild-type FAAH, individuating an IC_50_ value in the micromolar range (IC_50_ = 74 ± 8 μM) [186]. In the same year, Favia et al. docked more than 382 COX inhibitors in the structure of FAAH and identified carprofen as the starting point for the development of novel FAAH/COX inhibitors [187].

By installing various chemical modifications in the carprofen structure, such as the replacement of the chlorine atom, the conversion of the propanoic acid in different ester or amides or introducing functional groups on the carbazole nitrogen, new derivatives were obtained. In this SAR analysis, the pivotal role of propionic acid in the COXs inhibition was confirmed by the lack of activity of ester or amides analogues. However, the introduction of appropriate substituents on the carbazole nitrogen, combined with the presence of the chlorine atom gave two multitarget compounds, **6** (FAAH IC_50_ = 22.0 ± 4.2 μM, COX-1 IC_50_ = 74.3 ± 28.0 µM and COX-2 IC_50_ = 72.3 ± 28.0 µM) and **7** (FAAH IC_50_ = 84.8 ± 10.6 μM, COX-1 IC_50_ = 30.0 ± 12.2 µM and COX-2 IC_50_ = 27.8 ± 9.7 µM) reported in Table 4. Moreover, enantiomeric resolution for carprofen, **6** and **7** gave interesting results. Carpofen (*S*)-(*+*) enantiomer was the only compound active against all targets. According to literature data regarding other NSAIDs, (*R*)-(−) enantiomers of **6** and **7** (respectively, **6a** and **7a** in Table 4) turned out active against FAAH enzyme, while the corresponding (*S*)-(+) enantiomers were proved active only against COXs. In addition, carprofen, (*R*)-(−)-enantiomers of **6** and **7a** resulted completely inactive against COXs [187].

**Figure 2:**
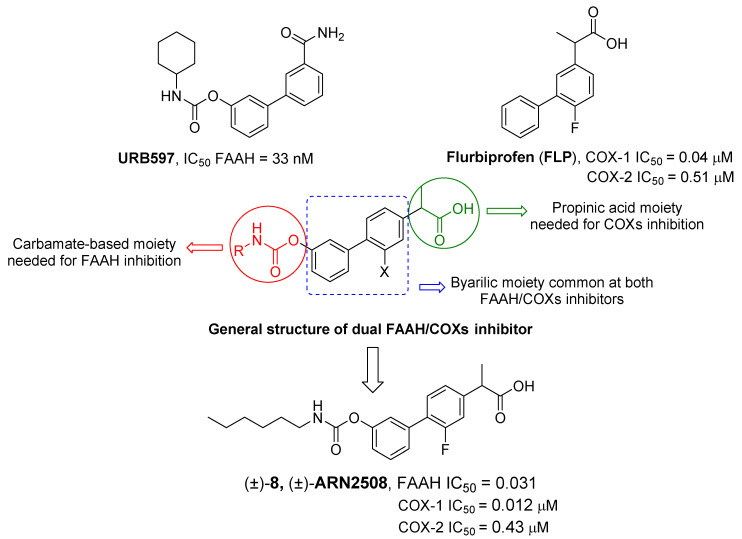
Representation of rational development of dual FAAH/COXs inhibitor (±)-ARN2508 (±-**8**) starting from FAAH inhibitor URB597 and COXs inhibitor flurbiprofen (FPL).

By combining key structural elements of the FAAH inhibitor URB597 and the 2-arylpropropionic acid COX1-2 ligand flurbiprofen (IC_50_ *h*COX-1 = 0.04 µM, IC_50_ *h*COX-2 = 0.51 µM) (FLP, Figure 2), Sasso et al. reported a class of multitarget FAAH-COX1-2 inhibitors [188]. Replacement of the biphenyl carbamate-based moiety of URB597 with the flurbiprofen scaffold, combined with an *N*-hexyl lateral chain, allowed to obtain the dual FAAH/COXs inhibitor ((±)-2-(3-fluoro-4-(3-(hexylcarbamoyloxy)phenyl)phenyl)propanoic acid ARN2508 (IC_50_ FAAH = 0.031 μM, IC_50_ COX-1 = 0.012 µM, IC_50_ COX-2 = 0.43 µM) (**8**, Figure 2).

In order to rationalize the binding mode of this lead compound, detailed molecular docking studies were performed. In the FAAH active site, compound **8** should establish a covalent bond with catalytic Ser-241, reproposing the same covalent inhibition as demonstrated for URB597 [188,189]. COXs noncovalent interactions were observed by molecular dynamic simulations, comparing the binding mode in the COX-1 binding pocket of AA, FLP and compound (±)-**8**. The propionic acid moiety of (±)-**8** was involved in an H-bond with the Arg120 confirming a typical binding mode of 2-arypropionic acid-based COXs inhibitors. The biphenyl groups of FLP and (±)-**8** occupied the same region in the COX-1 binding pocket, (±)-**8** established lipophilic interactions similarly to AA substrate [190,191]. Moreover, racemic administration of compound (±)-**8** (1, 3, 10, 30 mg/kg) showed anti-inflammatory effects in CD1 mice DSS-induced colitis, resulting more efficacious than mesalazine (5-ASA), currently used in the treatment of inflammatory bowel diseases. Aiming to identify new multitarget FAAH/COXs inhibitors, an in-depth SAR analysis was then performed starting from derivative (±)-**8** [192]. Replacing the propionic acid moiety with the achiral benzyl acid group, compound **9a** (FAAH IC_50_ = 0.063 ± 0.010 μM, COX-1 IC_50_ = 2.1 ± 0.1 µM and COX-2 IC_50_ = 0.24 ± 0.04 µM) was obtained (Table 5). This derivative turned out 2-fold less active against FAAH, 180-fold less active against COX-1 while the activity against COX-2 was slightly improved, compared to the reference compound (±)-**8**. The effects of the fluorine substitution with other groups were also explored highlighting that electronic and steric proprieties of the tested substituents did not play a relevant role in the FAAH recognition, while only lipophilic moiety allowed good interactions with COXs enzymes. Indeed, compounds **9b** (FAAH IC_50_ = 0.023 μM, COX-1 IC_50_ =0.009 µM and COX-2 IC_50_ = 0.73 µM), **9c** (FAAH IC_50_ = 0.010 μM, COX-1 IC_50_ = 0.011 µM and COX-2 IC_50_ = 1.40 µM), and **9d** (FAAH IC_50_ = 0.005 μM, COX-1 IC_50_ = 0.01 µM and COX-2 IC_50_ = 0.2 µM) (reported in Table 5), which bring, respectively, a chlorine, a methyl, and a trifluoromethyl group, showed a multitarget FAAH/COXs profile [192].

Additionally, the nature of the lateral chain was evaluated. Computational data justified the decreased multitarget activity derived from the replacement of *N*-hexyl moiety with aliphatic/aromatic ring or longer aliphatic chains. This can be due to an excessive steric hindrance, especially in the COX-2 binding pocket [188,191,192]. Enantiomeric resolution of compound (±)-**8** gave enantiomers (−)-**8** (FAAH IC_50_ = 0.0099 μM, COX-1 IC_50_ = 4.0 µM and COX-2 IC_50_ = 22.8 µM) and (+)-**8** (FAAH IC_50_ = 0.0094 μM, COX-1 IC_50_ = 0.00029 µM and COX-2 IC_50_ = 0.050 µM), reported in Table 5. Both enantiomers equally resulted more active against FAAH compared to the racemic mixture, while in discord with other studies, relevant differences were detected in the COXs inhibition potency. Compound (±)-**8** showed high potency against both COX enzymes, whereas its enantiomer (−)-**8a** was weakly active on either enzyme, compared to the racemic mixture [192].

#### 2.2.2. Hybrid FAAH/Soluble Epoxide Hydrolase (sEH) Inhibitors

**Figure 3:**
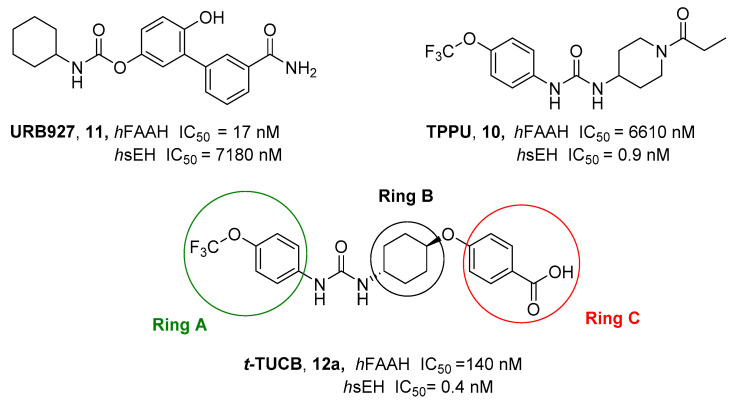
Chemical structures of the sEH inhibitors TPPU (**10**), FAAH inhibitor URB927 (**11**), *t*-TUCB (**12a**) and marked rings A, B, and C in the *t*-TUCB skeleton.

Soluble epoxide hydrolase (sEH) is a bifunctional enzyme which shows an unclarified phosphatase activity in the N-terminal domain, and a C-terminal hydrolase activity [193]. Lipidic mediators such as epoxidized fatty acids (EpFAs), involved in the pain modulation, are the endogenous substrates of the C-terminal sEH domain whose epoxide reactive moiety is converted in the corresponding dihydroxy fatty acid [193,194]. EpFAs resulted able to weakly active CBR_2_, selectively [195]. This partial activation of ECS EpFAS-mediated, combined to AEA-mediated effects, proposed a potential complementary and synergic activity of EpFAS and fatty acid ethanolamides (FAEs) in the pain modulation [178]. This complementarity was demonstrated by co-administration of sEP inhibitor 1-trifluoromethoxyphenyl-3-(1-propionylpiperidin-4-yl) urea TPPU (IC_50_ *h*FAAH = 6610 nM, IC_50_ *hs*EH = 0.9 nM, **10**, Figure 3) (0.03, 0.1, 0.3, 1, 3 mg/kg) with the peripherally restricted FAAH inhibitor URB937 (IC_50_ *h*FAAH 17 nM, IC_50_ *hs*EH = 7180 nM, **11** Figure 3) (0.03, 0.1, 0.3, 1, 3 mg/kg) in a mouse model of acute inflammation and in a rat model of neuropathy. Relevant synergic effects in the pain reduction were detected in both animal models [178]. Moreover, sEH inhibitor trans-4-[4-(3-trifluoromethoxyphenyl-l-ureido)-cyclohexyloxy]-benzoic acid t-TUCB (**12a**, Figure 3) showed a weak FAAH inhibitory potency (IC_50_ *h*FAAH = 140 nM). Starting from these data, Kodani et al. designed an innovative set of dual FAAH/sEH inhibitors merging the key structural elements needed to engage both enzymatic systems [196]. Several modifications on t-TUCB skeleton, respectively, in the ring A, B, and C (see Figure 3) were conducted to identify new dual compounds.

In the ring A, the 4-trifluorometoxy group of inhibitors **12a** were identified as a key moiety to maintain good potency against both targets. Switching the cyclohexyl group of the ring B in the cis conformation, a sensible decrease of activity against FAAH was detected. Whereas the conversion of the cyclohexane in an aromatic linker, allowed to obtain derivate **12b** (IC_50_ *h*FAAH 170 nM, IC_50_ *h*sEH = 7 nM), which showed an activity against FAAH comparable to that of the lead compound **12a** (Table 6). Linear linkers in the ring B were not well tolerated by the FAAH enzyme. At last, the benzoic acid moiety on the ring C was replaced with more lipophilic groups identifying the methyl ester **12c** (IC_50_ *h*FAAH 35 nM, IC_50_ *h*sEH = 7 nM), benzyl ester **12d** (IC_50_ *h*FAAH 24 nM, IC_50_ *h*sEH = 3 nM), and the methyl ester of the glycil amide **12e** (IC_50_ *h*FAAH 30 nM, IC_50_ *h*sEH = 3 nM) as derivatives with balanced FAAH/sEH inhibition properties (see Table 6). Studies on the mechanism of action clarified that these inhibitors act through a competitive mechanism against FAAH, since the inhibitory potency of **12a** and **12d** was not time dependent. The docking pose for compounds **12a** and **12c** within the FAAH active site showed that both the inhibitors interacted with the catalytic Ser241. Compound **12a** occupied the ACB pocket while compound **12d** stayed in the MAC [196].

Although these inhibitors resulted potent and selective against both enzymes, they showed a low species selectivity, indeed the best compound **12d** resulted less active in the FAAH enzyme from rodent species (*m*sEH IC_50_ = 5.7, *m*FAAH IC_50_ = 350 nM; *r*sEH IC_50_ = 54 nM, *r*FAAH IC_50_ = 1700 nM), thus becoming unsuitable for experiments in animals models [196]. With the aim to obtain dual FAAH/sEH inhibitors as suitable pharmacological tools for rodent in vivo models, the same authors designed novel compounds modifying the urea moiety. In particular, one urea nitrogen was directly connected to an aromatic leaving group, while the other nitrogen was bound to an heterocycle, such as piperidine or piperazine [197]. These structural manipulations allowed the facilitation of the nucleophilic attack of Ser241, thus enhancing the selectivity for the FAAH enzyme. The better results were obtained by modifying the leaving group of the potent FAAH inhibitor PF3845, thus leading to the dual inhibitors **13a**–**d** (Table 7) [197].

In general, the new derivatives resulted more potent against both *human* FAAH and sEH enzymes and against the corresponding *murine* and *rat* isoforms, compared to the previously reported analogues [196,197]. Moreover, after increasing the pre-incubation times (up to 1 h), the potency against rat and murine enzymes also increased. These results were consistent with an irreversible inhibition of the FAAH enzyme [197]. The ability to inhibit FAAH and sEH in vivo was also evaluated by detecting the residual enzymatic activity 4 h after i.p. injections of compound **13a** (10 mg/kg) in mice. Derivative **13a** was not effective in reducing FAAH activity (22% residual activity) and marginally reduced sHE activity in the brain. However, a dose response of **13a** also demonstrated > 60% FAAH inhibition at the lowest tested dose (1 to 100 mg/kg) [197].

#### 2.2.3. Hybrid FAAH/MAGL and Cholinesterase Inhibitors

Reduction of the cholinergic tone associated to memory and cognitive dysfunction, represents the main feature of AD [179]. Since the relevant role played by cholinergic neurons in this multifactorial disease, cholinesterase inhibitors (ChEIs) temporary improve clinical conditions by selective or dual inhibition of acetylcholinesterase (AChE) and butyrylcholinesterase (BuChE) [198,199]. Moreover, increased levels of IL-1, IL-6, and TNF-α, and reactive oxygen species (ROS) were detected in the brain of AD patients, indicate that neuroinflammatory processes are also involved in AD pathogenesis [200]. Therefore, the modulation of ECS can be a viable option to treat the neuroinflammation associated to AD. The viability of this new therapeutic prospect resides on the decreased levels of AEA observed in the brain of AD patients [201]. Accordingly, Rampa et al.reported the use of multitarget directed ligands modulating the endocannabinoid and the cholinergic systems as a potential and innovative therapeutic approach for this complex disease [179]. In a recent work, an in-house available library of carbamate-based ChEIs was tested against the FAAH enzyme for identifying the SAR needed to simultaneously engage the FAAH and the ChE enzymes. Experimental data suggested that the coumarin and the azaxanthone scaffold combined with a three-methylene chain were well tolerated by the FAAH enzyme, while a longer chain on N-substituent gave the better inhibition profile. Starting from this information, four new carbamates-based compounds were developed, characterized by FAAH and ChEs inhibition profile. Among these analogues, **14a** (IC_50_ *hr*FAAH = 2260 nM, IC_50_ *hr*AchE = 6647.9 nM, IC_50_ *hr*BuAchE = 1.57 nM, in Table 8), 14b (IC_50_ *hr*FAAH = 14,840 nM, IC_50_ *hr*AchE = 119 nM, IC_50_ *hr*BuAchE = 11.2 nM, in Table 8) bring coumaric scaffold, combined with *N*-phenylphentyl or *N*-hepthyl-morpholine lateral chain, respectively, while **14c** (IC_50_ *hr*FAAH = 520 nM, IC_50_ *hr*AchE = 89.5 nM, IC_50_ *hr*BuAchE = 1.71 nM, showed in Table 8) and **14d** (IC_50_ *hr*FAAH = 39,040 nM, IC_50_ *hr*AchE = 139 nM, IC_50_ *hr*BuAchE = 27.6 nM, Table 8) were azaxanthone derivatives, combined with the same two lateral chains.

**Figure 4:**
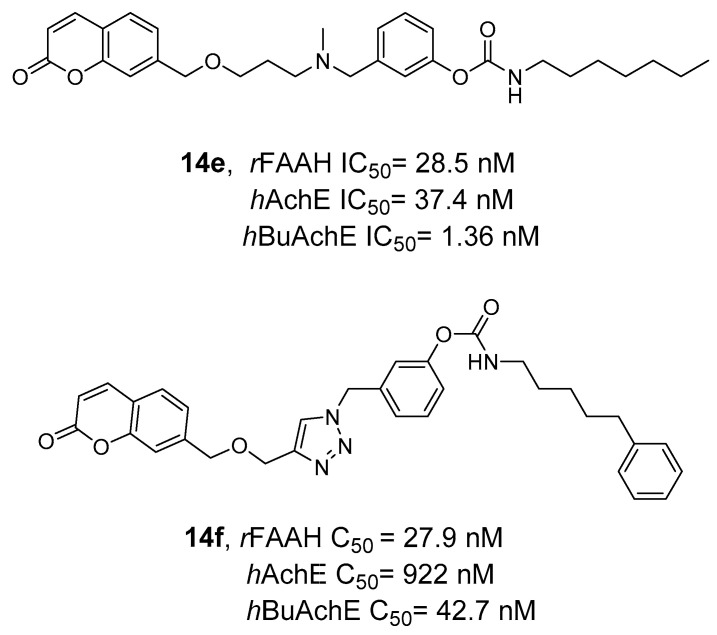
Chemical structure of FAAH/ChE inhibitors **14e** and **14f**.

All the new derivatives exhibited a nanomolar inhibition potency against the BuChE enzyme. Since the carbamate based ChEs inhibitors act as irreversible inhibitors, for these new compounds the binding mode against BuChE was also investigated. The nature of N-lateral chain plays a relevant role during the formation of a covalent adduct with BuAchE enzyme. In agreement with previous studies, the7-morpholinoheptyl derivative **14b** carbamoyled ChE enzymes more slowly than the phenylpentyl analogue **13**. Moreover, compound **14a** resulted more selective for the BuchE and together with compound **14c** also exhibited high potency against *r*FAAH enzyme. For these proprieties, compound **14a** and **14d** represented the early prototypes of dual FAAH/ChE inhibition [179]. Accordingly, the same authors, starting from the chemical structure of the lead compound **14a**, systematically analyzed the effects of N-chains length in the inhibition of AChE, BuChE, and FAAH. *N*-heptyl derivate **14e** (IC_50_ *hr*FAAH = 28.5 nM, IC_50_ *hr*AchE = 37.4 nM, IC_50_ *hr*BuchE = 1.36 nM, Figure 4) was identified as a potent inhibitor for all three selected targets. Kinetics studies of ChEs carbamoylation, performed on the derivate **14e**, evidenced as the N-heptyl lateral chain led to a faster inactivation of *h*BuChE compared to *h*AChE inhibition. These data demonstrated that the spacer-chain length did not influence the kinetics of *h*BuChE carbomoylation and explained the major inhibitory potency of **14e** against *h*BuChE. Moreover, molecular docking studies demonstrated that compounds **14e** and **14a** completely occupied the binding site of FAAH. The carbamate group was positioned very close to the Ser241, while the coumarin scaffold and the methylamine linker occupied the cytosolic port [202]. Notably, additional biological assays demonstrated that the IC_50_ against *h*FAAH was influenced by pH value employed during the in vitro test. Indeed, the activity against FAAH for compound **14a** was strongly affected by the pH conditions of the enzymatic test being decreased when operating at physiologic pH with respect to moderately basic pH (compound **14a** IC_50_ FAAH = 183.1 nM at pH = 9; IC_50_ FAAH = 4099.1 nM at pH = 7.4). These data indicated that probably the tertiary amine presents in the structure of compound **14a** should engage the FAAH binding site in its neutral state. To test this hypothesis, the amino-group of **14a** was replaced with a triazole ring, obtaining derivative **14f** (IC_50_ *hr*FAAH = 28.5 nM, IC_50_ *hr*AchE = 37.4 nM, IC_50_ *hr*BuchE = 1.36 nM, Figure 4) [202].

This latter compound showed higher activity against FAAH when compared to **14a** and **14e**, validating the proposed hypothesis. Compound **14f** was found to be a dual FAAH/BuChE inhibitor endowed with well-balanced nanomolar potency against both targets. In the FAAH catalytic pocket, compound **14f** proposed the same docking pose as identified for the lead compound **14a**. In the BuChE enzyme, the methylene spacer of compound **14f** allowed the access to BuChE gorge with the carbonyl group direct toward the catalytic triad (S198, H438 and E325). The phenyl ring attached to the carbamate group pointed toward the choline-binding pocket while the coumarin moiety occupied the wide access channel [202]. The selectivity profile of derivatives **14e** and **14f** designated these inhibitors as potential therapeutic tools for the treatment of moderate forms of AD, since with the progressive ChE reduction in the cholinergic neurons, BuChE becomes the main enzyme involved in the regulation of the cholinergic tone in the CNS [199,202]. In 2021, Rudolph et al. designed a library of N-(ω-indol-1-ylalkyl)-substituted phenyl carbamates as dual FAAH/BuChE inhibitors [203]. Starting from the general structure of indole-based FAAH inhibitors, a fluorine atom was inserted in the position 6 of the indole ring. The fluorine indole moiety, combined with a pentyl linker led to compound **15a** (IC_50_ *hr*FAAH = 0.029 µM, IC_50_ *hr*BuAchE = 4.3 µM, Table 9) which showed a dual FAAH/BuChE inhibition profile. Structure optimization of compound **15a** was then performed modifying opportunely the *O*-phenyl ring to increase the activity against FAAH and cholinergic enzymes. The reactive carbamate moiety of derivate **15b** (IC_50_ *hr*FAAH = 0.038 µM, IC_50_ *hr*MAGL = 0.038 µM, IC_50_ *hr*BuChE = 2.3 µM, Table 9) explained its equivalent activity against both cannabinoid catabolic enzymes and the good inhibitory potency against BuChE. Introducing an indol-4-yl carbamate moiety, the well-balanced dual FAAH/BuChE inhibitor **15c** (IC_50_ *hr*FAAH = 0.18 µM, IC_50_ *hr*BuAchE = 0.55 µM, Table 9) was obtained.

Due to the presence of a hydrolysable carbamate moiety, the chemical and the metabolic stability were evaluated, respectively, in aqueous solution and in porcine plasma. As predicted, the reactive carbamate **15b** showed a lower chemical and metabolic stability compared to derivate **15c**. For these reasons, indol-4-yl carbamate **15c** could be a lead compound in the development of dual FAAH/BuChE inhibitors for the treatment of AD [203].

#### 2.2.4. Hybrid FAAH/Dopaminergic System Modulators

In mesocorticolimbic areas, which play a key role in drug abstinence responses, ECS and the dopaminergic system are strongly connected [184]. Indeed, AEA activates dopaminergic conduction and the use of FAAH inhibitors increases dopaminergic tone [204]. Several pieces of evidence indicated that in smokers, D_2_/D_3_ receptors availability resulted noticeably decreased [205]. These data suggested that nicotine abuse can be treated with modulators of D_2_/D_3_ receptors or using multitarget FAAH/D_2_/D_3_ ligands [143]. In this context, De Simone et al.reported a small set of multitarget FAAH and D_3_ ligands, developed from an in silico library in which each molecule was characterized by chemical features needed to engage both targets. These molecules were docked into the crystal structures of rat FAAH and human D_3_ receptor and compounds **16a** and **16b** (Table 10) were predicted to be possible dual ligands. Biological data confirmed the proposed hypothesis. Compounds **16a**-**b** showed inhibitory activity against FAAH in the nanomolar range (IC_50_ FAAH = 0.3 nM and 1.3 nM, respectively) and at the same time they acted as D_3_ ligands (EC_50_ = 6.5 nM and 3.9 nM, respectively). However, these two inhibitors also showed a picomolar activity against CB_1_ receptor. The naphthyl group, owned by D_3_ ligands, was used to replace biphenyl-4-carboxamide group of compounds **16a**-**b**. Thus, the obtained compound **16c** (IC_50_ *h*FAAH = 6.1 nM, D3 EC_50_ = 1.3 nM, Table 10) resulted 450-fold less potent against CB_1_ receptors compared to prototype compounds **16a**-**b**. This new compound also showed a balanced activity against FAAH and D_3_ targets as well as a good selectivity versus D_2_ receptor [206].

**Figure 5:**
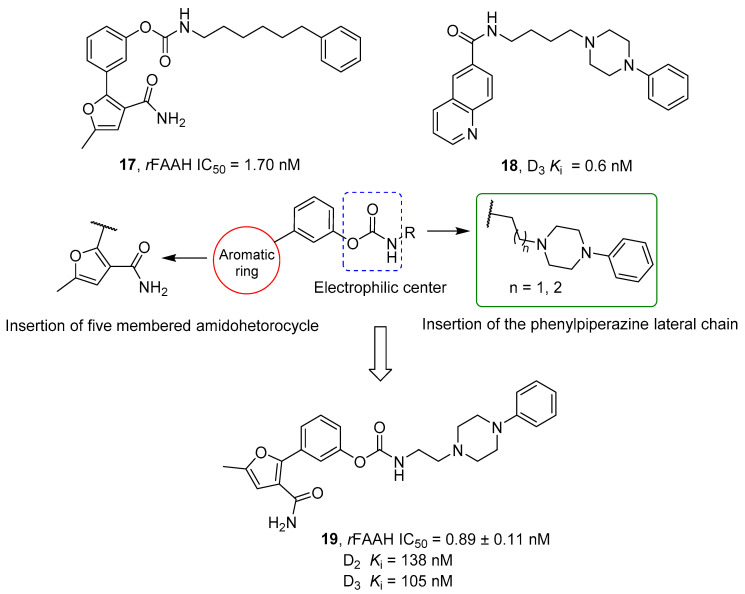
Representation of rational development of hybrid FAAH/dopamine receptor ligands **19,** starting from FAAH inhibitor **17** and the multitarget compound **18**.

In 2019, Grillo et al.taking inspiration from their previously reported FAAH inhibitors (compound **17**, Figure 5) [144] and D_2_/D_3_ multitargets ligands (compounds **18**, Figure 5) [207], designed a library of hybrid FAAH inhibitors and D_2_/D_3_ receptors ligands. Derivative **19** (IC_50_ *m*FAAH = 0.89 nM, *K*_i_ *h*D_2_ = 136 nM, *K*_i_ *h*D_3_ = 105, Figure 5) was identified as the lead compound in this new class of inhibitors for its balanced activity against all three targets [143].

Carbamate-based multitarget inhibitor **19** was obtained replacing the phenylhexyl lateral chain of the reference FAAH inhibitor whit a phenylpiperazine chain, inspired by compound **18**. This structural change was tolerated well by FAAH enzyme; at the same time, **19** was able to engage D_2_/D_3_ receptors subtypes. The docking pose of **19** in the FAAH binding pocket proved that the carbamate moiety was the key element to obtain a high FAAH inhibition potency. Urethane carbonyl group forms a polar contact with Ser241, while the amidic moiety of the phenylfurane scaffold interacts with S190, C269, and V270 backbones. The phenyl ring of the phenylpiperazine lateral chain establishes a triple π–π stacking with F192, F381, and F432 [143]. Docking studies were performed to rationalize the affinity of **19** for D_2_/D_3_ receptors subtypes. In D_2_ binding pocket, the phenylpiperazine chain was engaged in a series of π–π stacking and hydrophobic contacts with hydrophobic residues belonging to seven transmembrane helices. The biphenyl moiety establishes lipophilic contacts in the receptor pocket while the amide group forms an H-bond with the side chain of E95. In the D_3_ receptor, the binding mode of compound **19** turned out very similar to the D_2_ docking pose. Selectivity profile on **19** demonstrated that the lead compound did not interact with MAGL enzyme, CBRs and *h*ERG. Moreover, no relevant toxic effects were detected in murine fibroblast, astrocytes and in neuroblastoma cell line (IMR32) after administration of **19**. Since the increase in both pro-inflammatory cytokines and ROS production can be associated to nicotine abuse (promoting oxidative stress conditions) [208], anti-inflammatory profile of derivative **19** was also evaluated. Compound **19** significantly reduced LPS-induced activation of the redox-sensitive transcription factor NF-kB, in IMR 32 cell line [143].

#### 2.2.5. Hybrid FAAH/Melatonin Receptors Ligands

**Figure 6:**
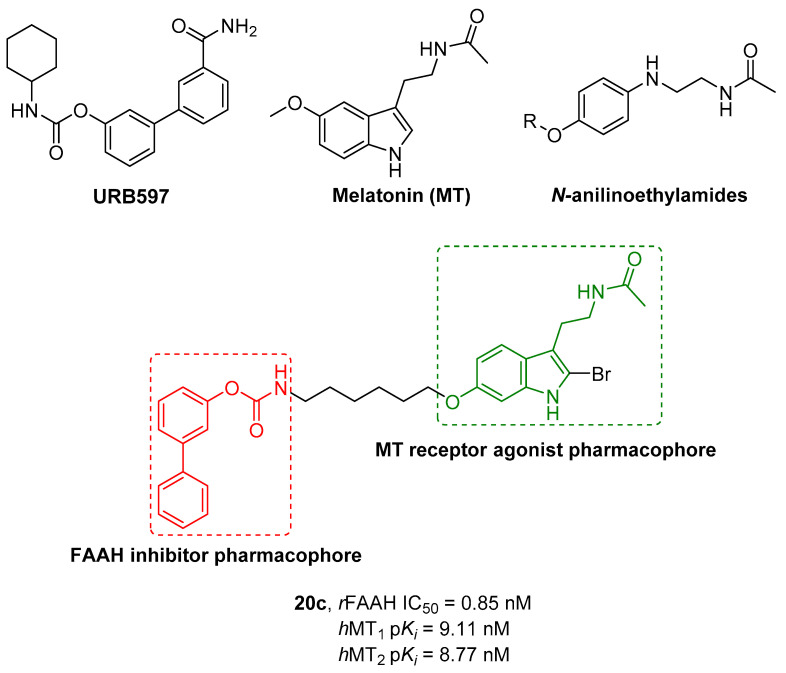
Chemical structures of FAAH and Melatonin receptor ligands.

WIN55212-mediated CBRs stimulation and activation of melatonin (MT, Figure 6) receptors (MT_1_ and MT_2_) showed to reduce intraocular pressure (IOP) leading to benefits in the glaucoma treatment [181,209,210]. Whereby, hybrid compounds able to activate both cannabinoid and melatonergic systems could represent an innovative pharmacological tool for the treatment of glaucoma, as reported by Spadoni et al. [181].

In this work, pharmacophoric elements of both the FAAH inhibitor URB597 and *N*-anilinoethylamides MT_1-2_ ligands were combined, obtaining multitarget compounds **20a** (*h*MT_1_ p*K*_i_ = 7.41 nM, *h*MT_2_ p*K*_i_ = 7.81 nM, *r*FAAH IC_50_ = 0.43 nM see Table 11), **20b** (*h*MT_1_ p*K*_i_ = 8.22 nM, *h*MT_2_ p*K*_i_ = 8.34 nM, *r*FAAH IC_50_ = 2.38 nM, see Table 11), and **20c** (*h*MT_1_ p*K*_i_ = 9.11 nM, *h*MT_2_ p*K*_i_ = 8.77 nM, *r*FAAH IC_50_ = 0.85 nM, see Figure 5 and Table 11) [181]. The cyclohexyl substituent of URB597 (Figure 5) was replaced with a linear alkyl chain, whose extremity was inserted an *N*-anilinoethilamide moiety for compound **20a**, and melatonin or melatonin bromidic derivate for compounds **20b** and **20c**, respectively. Derivative **20c** showed a balanced activity against all three targets and its docking pose in the FAAH binding pocket revealed several key interactions [181].

Carbamate moiety occupied the catalytic site, while the long *N*-lateral chain was accommodated in the ACB (Acyl chain binding) pocket. Whereas bromine atom of the 2-bromo indole moiety was involved in lipophilic interactions with Ala377, Leu380, Phe381, and Phe432. The activities of compounds **20a**–**c** were evaluated in a rabbit model of glaucoma in comparison with URB597, melatonin and the clinically used IOP lowering agent dorzolamide. At the concentration of 0.1 mM, compounds **20a** and **20c** give the best IOP lowering effect after 120, resulting more effective than the therapeutic agent dorzolamide. In light of these results, FAAH/melatonin ligands represent a valuable therapeutic alternative approach for treatment of ocular hypertension [181].

#### 2.2.6. Dual FAAH/TRPV1 Inhibitors

**Figure 7:**
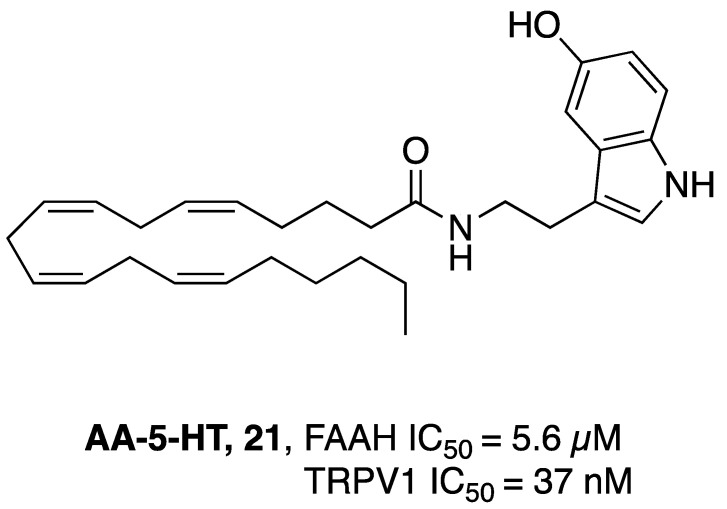
Chemical structure and IC_50_ values of AA-5-HT (21), against FAAH and TRPV1.

A fine tuning of the ECS and vanilloid system activity in the pantological conditions in which both systems are involved, represented a polypharmacological approach explored in the last few years. ECS play an important neuromodulatory role in the management of anxiety states. Indeed, the FAAH inhibitor URB597, directly injected in rat prefrontal cortex, reduces anxiety-like behaviors at low doses. However, high doses of URB597 in the same conditions showed opposite effects which were reversed by using TRPV1 antagonist [211]. This result suggests as the secondary AEA target, TRPV1, is involved in the modulation of anxiety. TRPV1 blockage in rats promotes anxiolytic-like effects while administration of TRPV1 agonists results in anxiogenic behavior [212]. In 2007, Maione et al. demonstrated that the FAAH inhibitor N-arachidinoyl-5-hydroxytrypatamine (AA-5-HT, 21, Figure 7) also showed an TRPV1 antagonist activity, identifying the prototype of dual FAAH-TRPV1 antagonist, potentially used as an analgesic agent [213].

As reported by Micale et.al, the dual inhibitor AA-5HT also resulted effective in controlling anxiety state in mice, confirming the opposite role of ECS and vanilloid system in the management of anxiety-like behaviors in mice [214]. Moreover, the administration of AA-5-HT in mice produced improvements in the forced swim test (FST), correlated to its effect on the hypothalamus–pituitary–adrenal axis which exerts a primary role in the etiology and progression of anxiety and depression state [215]. Remarkable alteration in the endocannabinoid and endovanilloid tone were found in null D3 receptor (D3R^−/−^) mice, which resulted less sensitive to anxiogenic and neurotoxic stimuli, compared to the wild-type. These data suggest that the crosstalk between ECS, dopaminergic system, and endovanilloid system is essential in establishing neuroprotective mechanisms [216].

## 3. Conclusions and Future Perspectives

The pharmacological treatment of complex and multifactorial pathologies such as neurodegenerative diseases, characterized by pathogenetic mechanisms involving numerous different pathways and neurotransmission systems, is still a paramount challenge. The ECS has attracted considerable attention as a potential therapeutic target for CNS and neurodegenerative diseases including PD, AD, HD, MS, ALS, stroke, TBI, pain, and epilepsy. The inhibition of FAAH and MAGL, the two major endocannabinoid hydrolyzing enzymes, has demonstrated to be an effective strategy in different preclinical models of these diseases, due to their anti-inflammatory, antioxidant, and neuroprotective effects, with lesser potential to cause side effects than the use of exogenous cannabinoid agonists. However, considering the multiple factors involved, a polypharmacological approach combining FAAH or MAGL with other pharmacological targets, could provide an edge to the treatment of CNS and neurodegenerative diseases. As reported in this review, numerous efforts have been made to develop FAAH inhibitors contextually interacting with other molecular targets. On the other hand, apart from hybrid dual FAAH/MAGL inhibitors, no other examples of the involvement of MAGL enzymes in polypharmacological approaches are reported in literature. Probably, the complexity of MAGL pharmacophore enhances the difficulty for a merging of the key structural elements needed to obtain potential multitarget compounds. Moreover, this challenging rational design could also make impervious the chemical synthesis; thus, discouraging the development of MAGL-based polypharmacological derivatives. Although still difficult at the current stage, it is extremely desirable, in the near future, to design and develop multitarget agents that simultaneously address endocannabinoid hydrolyzing enzymes and the most significant pathological pathways involved in CNS and neurodegenerative diseases.

## Figures and Tables

**Table 1:** Activity of racemic compounds (±)-**1** and (±)-**2** and their respective enantiomers against *hr*MAGL and 2-OG and AEA hydrolysis inhibition.

General Structure A						General Structure B					
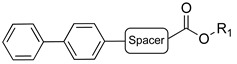						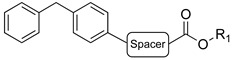					
Cpds	Spacer	R_1_	Hydrolysis Inhibition (µM)			Cpds	Spacer	R_1_	Hydrolysis Inhibition (µM)		
			*hr*MAGL	2-OG	AEA				*hr*MAGL	2-OG	AEA
(±)-**1**	(CH_2_)_5_	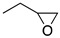	4.31	1.8	5.1	(±)-**2**	(CH_2_)_2_	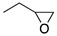	16	10	0.28
(*R*)-**1a**	(CH_2_)_5_	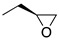	33 ± 5%	4.9	3.9	(*R*)-**2a**	(CH_2_)_2_	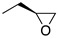	2.4	0.68	0.29
(*S*)-**1b**	(CH_2_)_5_	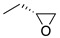	(45 ± 5%) ^a^	5.1	4.5	(*S*)-**2b**	(CH_2_)_2_	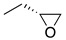	n.i ^b^	70	0.34

^a^ Percentage of inhibition attained at 10 or 100 μM; ^b^ n.i. indicates < 10% inhibition at the highest concentration tested (10 μM for *hr*MAGL or 100 μM for 2-OG or AEA hydrolysis).

**Table 2:** IC_50_ values on FAAH and MAGL enzyme for phenoxyphenyl piperazinyl and methylene-3,4-dioxyphenyl piperidinyl derivatives.

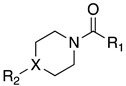					
Cpds	R_1_	R_2_	X	MAGL IC_50_ (nM) ^a^	*hr*FAAH IC_50_ (nM) ^b^
**3a**	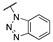	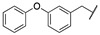	N	7.8	89
**3b**	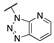	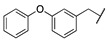	N	5.5	23
**3c**	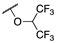	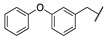	N	74	76 ^c^
**3d**	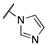	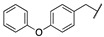	N	660	3.4
**3e**	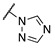	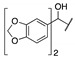	CH	0.7	622

^a^ Human recombinant MAGL expressed transiently in HEK293 cells. ^b^ Human recombinant FAAH expressed transiently in COS7 cells. ^c^ Remaining activity at 10 µM (% control).

**Table 3:** IC_50_ values on FAAH and MAGL enzyme for pyrroloquioxaline-based dual inhibitors.

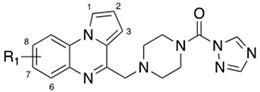			
Cpds	R_1_	MAGL ^a^ IC_50_ (nM)	FAAH ^b^ IC_50_ (nM)
**4a**	H	37.0	44.7
**4b**	7-F	10.7	49.9
**4c**	7-Cl	32.4	95.5
**4d**	7,8-diMe	32.4	80.1

^a^ rat brain membrane; ^b^ COS cells cytosol.

**Table 4:** FAAH, COX-1, and COX-2 activities of racemic and single enantiomers of carprofen, compounds **6** and **7**.

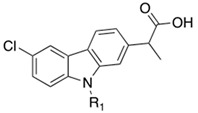				
Compounds	R_1_	IC_50_ (µM) ± SD		
		FAAH	COX-1	COX-2
Carprofen	H	76.6 ± 19.7	22.3 ± 6.6	3.9 ± 1.0
(*S*)-(+)–Carprofen	H	64.2 ± 3.6	5.6 ± 0.1	5.3 ± 3.0
(*R*)-(−)–Carprofen	H	>100	>100	>100
(±)-**6**	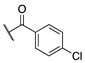	22.0 ± 4.2	74.3 ± 28.0	72.3 ± 28.0
(*R*)-(−)–**6a**	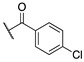	14.9 ± 1.6	>100	>100
(*S*)-(+)–**6b**	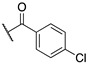	>100	45.0 ± 0.3	46.5 ± 4.3
(±)-**7**	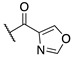	84.8 ± 10.6	30.0 ± 12.1	27.8 ± 9.7
(*R*)-(−)–**7a**	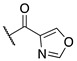	53.2 ± 22.6	>100	>100
(*S*)-(+)–**7b**	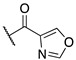	>100	4.1 ± 2.8	2.5 ± 1.4

**Table 5:** FAAH, COX-1, and COX-2 activities of racemic compounds (±)-**8**, **9a**–**d**, and single enantiomers compounds (−)-**8**, and (+)-**8**.

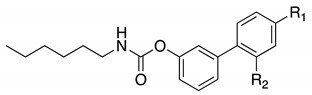					
Cpds	R_1_	R_2_	IC_50_ (µM) ± SD		
			FAAH	COX-1	COX-2
(±)-**8**	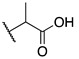	-F	0.031 ± 0.002	0.012 ± 0.002	0.43 ± 0.025
**9a**	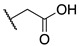	-F	0.063 ± 0.010	2.1 ± 0.1	0.24 ± 0.04
**9b**	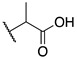	-Cl	0.023 ± 0.008	0.009 ± 0.001	0.73 ± 0.21
**9c**	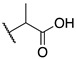	-CH3	0.010 ± 0.001	0.011 ± 0.001	0.40 ± 0.31
**9d**	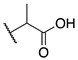	-CF_3_	0.005 ± 0.001	0.01 ± 0.003	0.2 ± 0.08
(−)-**8**	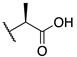	-F	0.0099 ± 0.002	4.0 ± 1.3	22.8 ± 8.7
(+)-**8**	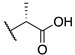	-F	0.0094 ± 0.003	0.00029 ± 0.0004	0.050 ± 0.012

**Table 6:** FAAH and sEH activities of *t*-TUCB derivatives.

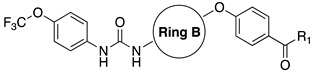						
Cpds	Ring B	R_1_	IC_50_ (nM)			
			*h*FAAH	*h*sEP	*m*FAAH	*r*FAAH
***t*-TUCB** (**12a**)	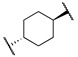	-OH	0.8	140	-	-
**12b**	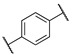	-OH	7	170	n.t	n.t
**12c**	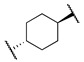	-OMe	7	35	n.t	n.t
**12d**	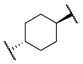	-OBn	3	24	510	>10,000
**12e**	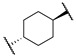	-NHCH_2_CO_2_Me	3	30	n.t	n.t

n.t.: not tested.

**Table 7:** FAAH and sEH activities of **PF3845** derivatives.

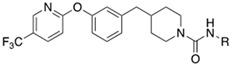							
Cpds	R	IC_50_ (nM)					
		*h*FAAH ^a^	*h*sEP ^a^	*m*FAAH ^a^	*m*FAAH ^b^	*m*FAAH ^a^	*r*FAAH ^b^
**13a**	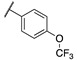	8	5	1400	66	>10,000	330
**13b**	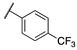	8	7	>10,000	290	>10,000	710
**13c**	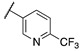	3	60	560	28	>10,000	110
**13d**	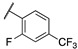	3	9	>10,000	340	>10,000	1100

^a^ 5 min preincubation time. ^b^ 60 min pre incubation time.

**Table 8:** Inhibitory activities of newly designed compounds against FAAH and human ChEs.

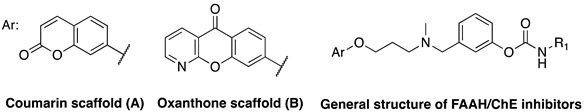							
Cpds	Ar	R_1_	IC_50_ (nM)				
			*r*FAAH ^a^	*r*FAAH ^b^	*hr*FAAH ^b^	*hr*AChE	*hr*BuChE
**14a**	A	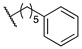	280	50	2260	6647.9	1.57
**14b**	A	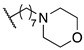	5590	1820	14,840	119	11.2
**14c**	B	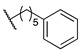	370	40	520	89.5	1.71
**14d**	B	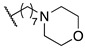	4310	2710	39,040	139	27.6

^a^ no preincubation time, ^b^ 20 min pre-incubation time.

**Table 9:** Inhibitory activities against human recombinant FAAH, MAGL, and ChEs and chemical and metabolic stability in aqueous PBS Buffer and porcine blood plasma.

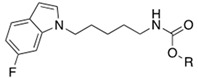						
Cpds	R	IC_50_ (µM)			Stability (%) ^a^	
		*hr*FAAH	*hr*MAGL	*hr*BuChE	PBS Buffer (pH 7.4)	Porcine Blood Plasma
**15a**	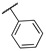	0.029	n.a.	4.3	>95	83 ± 9
**15b**	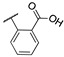	0.038	0.038	2.3	73 ± 6	28 ± 4
**15c**	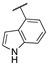	0.18	n.a.	0.55	>95	80 ± 3

n.a. not active at 10 µM; ^a^ Percentage of remaining compound after incubation in porcine plasma, or PBS buffer (pH 7.4).

**Table 10:** Inhibitory activities against rat and human FAAH, D_3,_ and D_2_ dopamine receptors, and CB_1_ receptors.

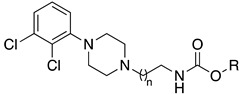							
Cpds	n	R	*r*FAAH IC_50_ (nM)	*h*FAAH IC_50_ (nM)	D_3_ EC_50_ (nM)	D_2_ EC_50_ (nM)	CB_1_ EC_50_ (nM)
**16a**	2	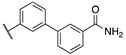	0.3	1.6	6.5	>1000	0.9
**16b**	1	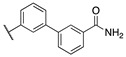	0.1	1.3	3.9	240.0	0.3
**16c**	2	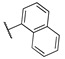	22.0	6.1	1.3	209.0	420.0

**Table 11:** Inhibitory activities of compounds **20a–c** against *h*MT_1-2_ receptors and *r*FAAH.

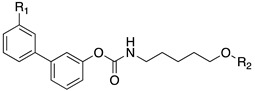					
Cpds	R_1_	R_2_	*h*MT_1_ p*K*_i_ ± SD	*h*MT_2_ p*K*_i_ ± SD	*r*FAAH IC_50_ (nM) ± SD
**20a**	-CONH_2_	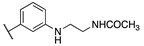	7.41 ± 0.03	7.81 ± 0.05	0.43 ± 0.01
**20b**	-H	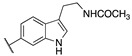	8.22 ± 0.01	8.34 ± 0.09	2.38 ± 0.16
**20c**	-H	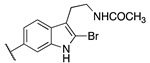	9.11 ± 0.10	8.77 ± 0.03	0.85 ± 0.01

## Supplementary Materials

The following supporting information can be downloaded at: https://www.mdpi.com/article/10.3390/cells11030471/s1, Figure S1: Chemical structures of FAAH, MAGL, and cannabinoid receptor ligands.
