# Supplementary figures and images for: Polypharmacological Approaches for CNS Diseases: Focus on Endocannabinoid Degradation Inhibition
Source: Cells. 2022 Jan 29;11(3):471. doi: 10.3390/cells11030471 (PMC8834510; doi:10.3390/cells11030471)

**Figure S1.** Chemical structures of FAAH, MAGL, and cannabinoid receptor ligands.

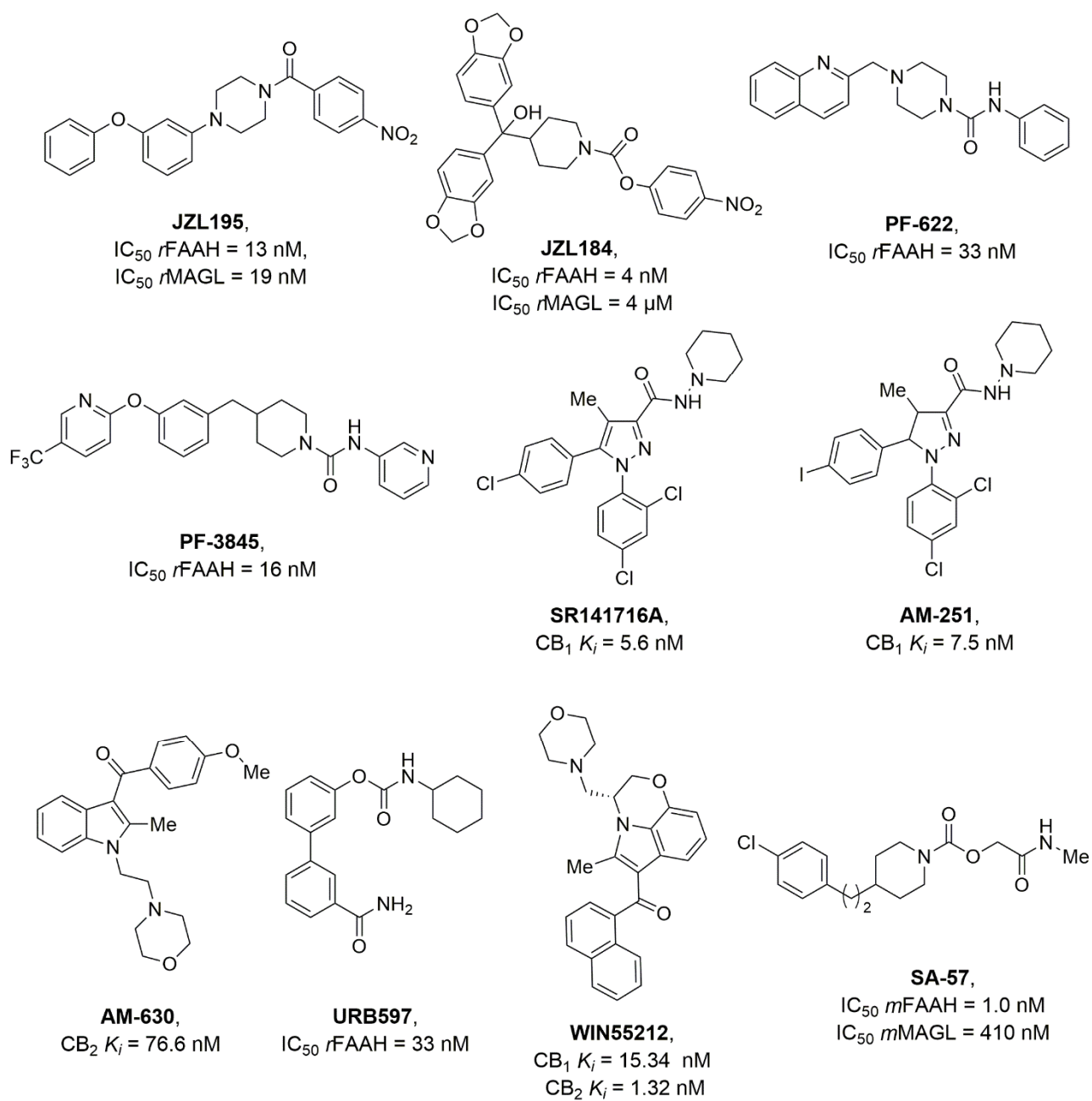

Supplement: Supplementary file 1 [file cells-11-00471-s001.zip › cells-1546996-supplementary.pdf]
